# Differences in Highly Pathogenic H5N6 Avian Influenza Viral Pathogenicity and Inflammatory Response in Chickens and Ducks

**DOI:** 10.3389/fmicb.2021.593202

**Published:** 2021-01-29

**Authors:** Bo Wang, Qianqian Su, Jing Luo, Meng Li, Qiaoxing Wu, Han Chang, Juan Du, Chengmei Huang, Jiajun Ma, Shuyi Han, Guohui Yuan, Yapeng He, Minglei Guo, Qingxun Zhang, Hongxuan He

**Affiliations:** ^1^National Research Center for Wildlife-Borne Diseases, Institute of Zoology, Chinese Academy of Sciences, Beijing, China; ^2^College of Life Science, University of Chinese Academy of Sciences, Beijing, China

**Keywords:** H5N6, phylogenetic analysis, chickens and ducks, pathogenic analyses, inflammatory response

## Abstract

Infection with H5N6 highly pathogenic avian influenza virus caused high mortality in chickens, while ducks often appear to be asymptomatic. But, some recent H5Nx subtype viruses could cause high mortality in ducks. The variation between different species and the mechanisms by which some H5Nx viruses cause death in ducks requires investigation to identify the key processes in influenza susceptibility and pathogenesis. Here, we characterized two representative H5N6 viruses, A/*Pavo cristatus*/Jiangxi/JA1/2016 (JA1) and A/*Anas crecca*/shanghai/SH1/2016 (SH1), and compared their pathogenicity and expression profiles of immune-related genes in chickens and ducks to identify the elements of the host immune-related response that were involved in disease lethality. Results suggested that H5N6 HPAIVs had higher pathogenic and inflammatory effect in chickens than in ducks. Importantly, the TNF-α, IL-6, IFN-γ and iNOS levels were significantly higher in the lung of SH1 infected chickens compared to those of ducks. And we found higher systemic levels of IL-6 induced by JA1 in chickens than in ducks. In addition, our experiments demonstrated that JA1 was associated with greater pathogenicity in ducks were accompanied by the excessive expression of iNOS in the brain. These results are helpful to understand the relationship between the pathogenicity of H5N6 AIVs and inflammatory responses to them in chickens and ducks.

## Introduction

Influenza viruses that originate from birds or swine have led to four pandemics since 1918. Seasonal influenza viruses, such as H1N1 and H3N2, result in 250,000–500,000 deaths annually worldwide (Krammer et al., 2018). In addition, highly pathogenic avian influenza viruses (HPAIVs, including H7N9, H5N1, and H5N6 subtypes) pose an ongoing threat to the animal and human health (Su et al., 2017). The extraordinary ability of influenza A viruses (IAVs) to mutate, resort, and recombine during spillover gives IAVs a potential to cause another influenza pandemic (Dunning et al., 2020).

Migratory waterfowls in the Orders Anseriformes (mainly ducks, geese, and swans) and Charadriiformes (mainly gulls, terns, and waders) are considered as the natural reservoir for all known subtypes (H1-H16 and N1-N9) of IAVs (Gilbert et al., 2006; Neumann et al., 2010; Huang et al., 2013). Infection with HPAIVs, such as H5N1, H5N6, and H5N8, in waterfowls is often asymptomatic, but in chickens and in humans, it can induce severe disease symptoms and death (Bean et al., 2013; Burggraaf et al., 2014; Kwon et al., 2018b; Uchida et al., 2019). However, some recent H5Nx subtype viruses caused unusually high mortality in ducks (Sturm-Ramirez et al., 2004; Li et al., 2017a; Kwon et al., 2018a; Leyson et al., 2019; Horwood et al., 2020). Comparative analyses between natural hosts (e.g., ducks) and spillover hosts (e.g., chickens) and understanding the mechanisms by which some H5Nx viruses cause death in ducks will help to identify the key processes in influenza susceptibility and pathogenesis.

The lethal effect of virus-induced inflammation is mediated partly by host responses to viral infection rather than being a direct cytopathic consequence of viral replication (Akaike et al., 2003). The innate immune system detects viral particles by using pattern recognition receptors (PRRs) that recognize pathogen associated molecular patterns (PAMPs; Abbas et al., 2017). There are members of at least three different classes of PRRs that are involved in the detection of IAVs: Toll-like receptor (TLR) family members, TLR3, TLR7, and TLR8; retinoic acid-inducible gene (RIG)-like receptor (RLR) family members, retinoic acid-inducible gene I (RIG-I) and MDA5; and the nucleotide-binding oligomerization domain (NOD)-like receptor (NLR) family member, NOD-, LRR-, and pyrin domain-containing 3 (NLRP3; Coates et al., 2015). Activation of these PRRs induces the secretion of cytokines, including interferons (IFNs), interleukins (IL), lymphokines, and tumor necrosis factors (TNF), which are the key modulators of inflammation (Iwasaki and Pillai, 2014). In addition, NO is an inflammatory mediator, which is generated by NO synthase (NOS; Ying et al., 2013). The inducible NO synthase (iNOS) is an inducible enzyme and an indicator of NO in the inflamed tissue (Yang et al., 2009; Burggraaf et al., 2011). After infection with HPAIVs, such as H5N1 and H7N1, waterfowl develop a limited inflammatory response, usually with little or no mortality, but chickens display a rapid and strong inflammatory response and the infection can induce severe disease symptoms (Bean et al., 2013; Cornelissen et al., 2013; Burggraaf et al., 2014). Innate-immune related genes were involved in the chickens and ducks innate immune response to H5N6 HPAIVs infection (Gao et al., 2017; Wu et al., 2019), but the difference in the inflammatory response to H5N6 between chickens and ducks and the pathogenic relevance of cytokine are unknown.

Here, we performed genetic and phylogenetic analysis of two H5N6 HPAIVs isolated from southern China, 2016. By extension, we compared the pathogenicity and transmissibility of the two H5N6 viruses in chickens and ducks. Further, we evaluated the expression profile of PRRs, proinflammatory cytokines and inflammatory mediator in chickens and ducks after infection with H5N6 virus.

## Materials and Methods

### Virus Isolation and Sequencing

Two H5N6 HPAIVs were used for the animal studies. A novel H5N6 virus, termed as A/*Anas crecca*/shanghai/SH1/2016 (SH1), was isolated from an apparently healthy wild duck (*Anas crecca*) during our active surveillance of AIVs in November 2016, in Shanghai Province, China. Another H5N6 isolate, namely A/*Pavo cristatus*/Jiangxi/JA1/2016 (JA1), stored in our lab, was isolated from dead peafowls (*Pavo cristatus*) in a breeding farm in February 2016, in Jiangxi Province, China. For virus isolation, the supernatants of swab samples were inoculated into 9–10-day old specific-pathogen-free (SPF) embryonated chicken eggs. The 50% egg infective doses (EID_50_) of the harvested allantoic fluids were determined by the method of Reed and Muench (Kang et al., 2018). Viral RNA was extracted from allantoic fluids using TRIzol Reagent (Invitrogen), and reverse transcribed with the primer Uni12 5'-AGCRAAAGCAGG-3' and GoScript™ Reverse Transcriptase System (Promega). We applied a multisegment reverse transcription PCR (M-RTPCR) approach to simultaneously amplify eight genomic RNA segments (Zhou et al., 2009). Genomic sequences were deposited into the GenBank (GenBank accession numbers: MH022713–MH022720).

### Phylogenetic Analysis

Phylogenetic trees were generated based on top BLAST hits in Sharing All Influenza Data (GISAID) and National Centre for Biotechnology Information (NCBI), in addition to the H5 clade classification reference sequences recommended by World Health Organization (WHO), World Organization for Animal Health (OIE), and Food and Agriculture Organization of the United Nations (FAO) guidelines (Bi et al., 2016). We used JModeltest2 to choose the best-fit model of nucleotide substitution (Drummond and Bouckaert, 2015). The relaxed molecular clock models were used to estimate divergence times. The Markov chain Monte Carlo (MCMC) chains were run for 100–200 million iterations. The best fit substitution models, relaxed molecular clock models, and tree models are listed in Supplementary Table S1. We checked the convergence by computing effective sample sizes (ESS > 200; Zhang et al., 2018). Maximum clade credibility (MCC) trees were constructed using Tree Annotator v1.8.4.

### Animals Infection Experiments

Thirty-two 6-week-old SPF white leghorn chickens and 32 8-week-old mallards seronegative for H5 were used (Supplementary Tables S1, S2). Sixteen birds were intranasally inoculated with 0.2 ml 10^6.5^ EID_50_ of either SH1 or JA1 virus (Supplementary Tables S2, S3). A control group (*n* = 12) was intranasally inoculated with 0.2 ml of phosphate buffered saline (PBS). After 24 h, four naïve birds (as physical contact) were introduced into the same cages with inoculated birds (Pantin-Jackwood et al., 2016). At 2 days post-infection (DPI), three birds from each treatment and control group were humanely euthanized to evaluate histopathologic changes, viral loads, and cytokine profiles in heart, liver, spleen, lung, kidney, pancreas, stomach, intestine, brain, and trachea tissues. The remaining birds were monitored daily for clinical symptoms and weight changes for 14 days. At 1, 2, 3, 4, 7, and 14 DPI, oropharyngeal and cloacal swabs samples were taken for the test of viral shedding. Seroconversion of all surviving birds at 14 DPI was confirmed by the hemagglutination inhibition (HI) assays using homologous antigens and 1% suspension of chicken red blood cells (Kang et al., 2018). The HI titers were described as reciprocal log2. Samples with a titer of eight or higher considered positive (Pedersen, 2008). Tissues were fixed and stained using hematoxylin and eosin (H&E) staining for histopathologic analysis. The sections were incubated with primary antibodies against nucleoprotein (GTX125989, Genetex) for immunohistochemical study. For quantification, 15 images were randomly selected from each group by pathologist in a blinded manner, and the intensity of staining was analyzed using Image J software.

### Real-Time Polymerase Chain Reaction (qPCR)

Total RNA was extracted from swab and tissue samples using Trizol Reagent (Invitrogen). The RNA (2 μg) was reverse-transcribed using primer Uni12 and oligo-dT and the GoScript™ Reverse Transcriptase System (Promega, A5004). Quantitative real-time polymerase chain reactions (qRT-PCRs) targeting the influenza virus M gene were conducted using UltraSYBR Mixture (Cwbio, CW2601M) and the ABI 7500 Fast Real-Time PCR system (Applied Biosystems, Carlsbad, CA). For absolute quantification, two standard curves were generated using 10-fold dilutions of two H5N6 HPAIVs of known titer, respectively, on ABI 7500 Fast Real-Time PCR system according to manufacturer instructions. Alternatively, tissue and swab samples from infected avian were quantified by real-time RT-PCR to determine the viral RNA yield. The results were extrapolated from corresponding standard curves and expressed as EID50/g or EID50/ml equivalents (Delang et al., 2014; Spackman, 2014). The detection limits for both viruses were 10^2.7^ EID_50_ (CT = 35; Pantin-Jackwood et al., 2016). Expression of immune-related genes was analyzed by qRT-PCR. Primers are shown in Supplementary Table S4. Target gene expression were normalized to the reference endogenous glyceraldehyde 3-phosphate dehydrogenase (GAPDH) gene and calculated using the 2-ΔΔCT method (Livak and Schmittgen, 2001; Guo et al., 2020).

### Statistical Analyses

Statistical analysis was performed either by two-way analysis of variance (ANOVA) or by application of Student’s *t*-test using GraphPad Prism version 8.0 software; *p* < 0.05 was considered as significant. The error bars represent the SD. Birds and their analyzed features were clustered using the hierarchical clustering by computing Spearman’s coefficients. Heat maps were created using the ComplexHeatmap package (Lucas et al., 2020).

### Biosafety Facility and Ethics Statement

We performed all experiments involving the HPAIVs in an Animal Biosafety Level (ABSL) 3 containment laboratory approved by the Chinese Academy of Sciences. All animal studies were approved by the Committee on the Ethics of Animal Experiments in the Institute of Zoology, Chinese Academy of Sciences (approval number: IOZ-15042).

## Results

### Characterization of the Two H5N6 Viruses Isolated From Southern China in 2016

Putative amino acid sequences were compared between the two H5N6 influenza viruses, JA1 and SH1, to reveal any relationship between pathogenicity in chicken duck and the amino acid sequences of the viruses (Table 1; Supplementary Table S11). Both of them possessed multiple basic amino acids (-RERRRKR↓GLF-) at the hemagglutinin (HA) cleavage site, characteristic for HPAIVs (Table 1; Subbarao et al., 1998). The Q222L and G224S substitutions in receptor binding domain of HA (H5 numbering system) suggested preference for the avian-type receptor [sialic acid alpha-2,3-galactose (SAα2,3Gal); Matrosovich et al., 2000]. Importantly, JA1 contains avian-like NS1 C-terminal PDZ domain ligand (PL) residues of ESEV, while SH1 has an NS1 protein with a C-terminal four-residue PL sequence of ESEI. Interestingly, the PB1-F2 protein is N-terminally truncated in JA1 (52 aa), although SH1 encode a full-length protein (99 aa).

**Table 1:** Molecular characterization of SH1 and JA1.

Gene	Phenotype/Function	Mutations	SH1	JA1
	Highly pathogenic Cleavage peptides		RERRRKR↓G	RERRRKR↓G
HA^a^	Receptor-binding specificity	Q222L	Q	Q
		S223N	Q	R
		G224S	G	G
NA	Increase the virulence to mammals	59–69 del	Yes	Yes
PB2	Increased transmission in mammals	E627K	E	E
		D701N	D	D
PB1-F2	Influence pathogenicity in mice	2–39 del	No	Yes
PA	Increased transmission	S409N	N	S
		672L	L	L
NS1	Increased virulence in mice	227–230 ESEV	ESEV	ESEI
NS2	Increased virulence in chickens and mice	80–84 del	Yes	Yes

Del: deletion; aa: amino acid.

a H5 numbering.

**Figure 1:**
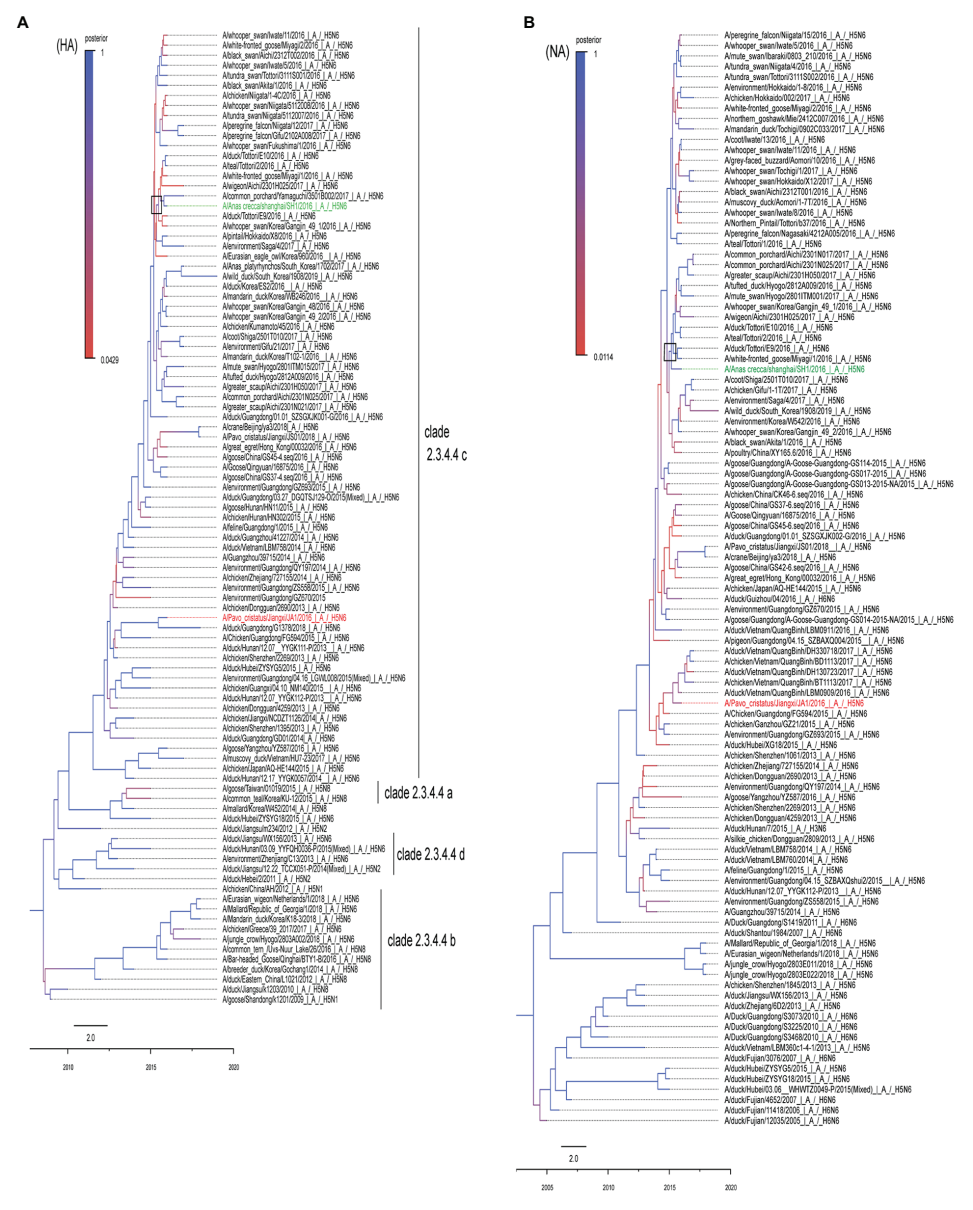
MCC trees of HA and HA genes of SH1 and JA1 viruses. The phylogenetic analyses were conducted using the listed gene sequences, which were obtained from the GISAID and NCBI GenBank database. Panels **(A,B)** represent the MCC trees of HA and NA genes, respectively. SH1 is marked in green and JA1 is marked in red. The trees were built by BEAST software (V1.8.4) and illustrated by Fig Tree (v1.4.3). Time-scaled phylogenies (date-based axis shown at the bottom of the figure) were inferred using uncorrelated relaxed clock Bayesian Markov chain Monte Carlo (MCMC) analysis. HA, hemagglutinin and NA, neuraminidase.

To clarify the phylogenetic relationship of the two H5N6 viruses, we compared their eight gene segments with sequences of typical influenza viruses obtained from GISAID and the NCBI GenBank database. Homology analysis suggested the homologies of the eight gene segments of the two virus strains are 98% (polymerase basic protein 2, PB2), 89% (polymerase basic protein 1, PB1), 93% (polymerase acidic, PA), 97% (hemagglutinin, HA), 98% (nucleoproteins, NP), 99% (neuraminidase, NA), 99% (matrix, M), and 98% (nonstructural gene, NS), indicating that both viruses most likely obtained their NA and M genes from a recent common ancestor. The resulting MCC trees revealed that JA1 and SH1 HA genes were clustered into the H5 clade 2.3.4.4 (Figure 1A), whereas their NA genes belonged to the Eurasian lineage (Figure 1B). A previous study indicated that a minimal amount of the identified H5N6 isolates belonged to the North American lineage, and the N6 genes of H5N6 AIVs were not transmitted to other continents (Li et al., 2017b). The MCC trees based on eight gene segments showed that JA1 shared the highest homology with A/Chicken/Guangdong/FG594/2015 (H5N6; Li et al., 2017b), while SH1 shared the highest homology with A/duck/Tottori/E9/2016 (H5N6; Figure 1; Supplementary Figure S1; Supplementary Table S5). In addition, SH1 belonged to the C6 genotype, which carried PA-III and NS-I, according to the previous research (Takemae et al., 2017).

### Pathogenicity of the Two H5N6 Viruses in Chickens and Ducks

**Figure 2:**
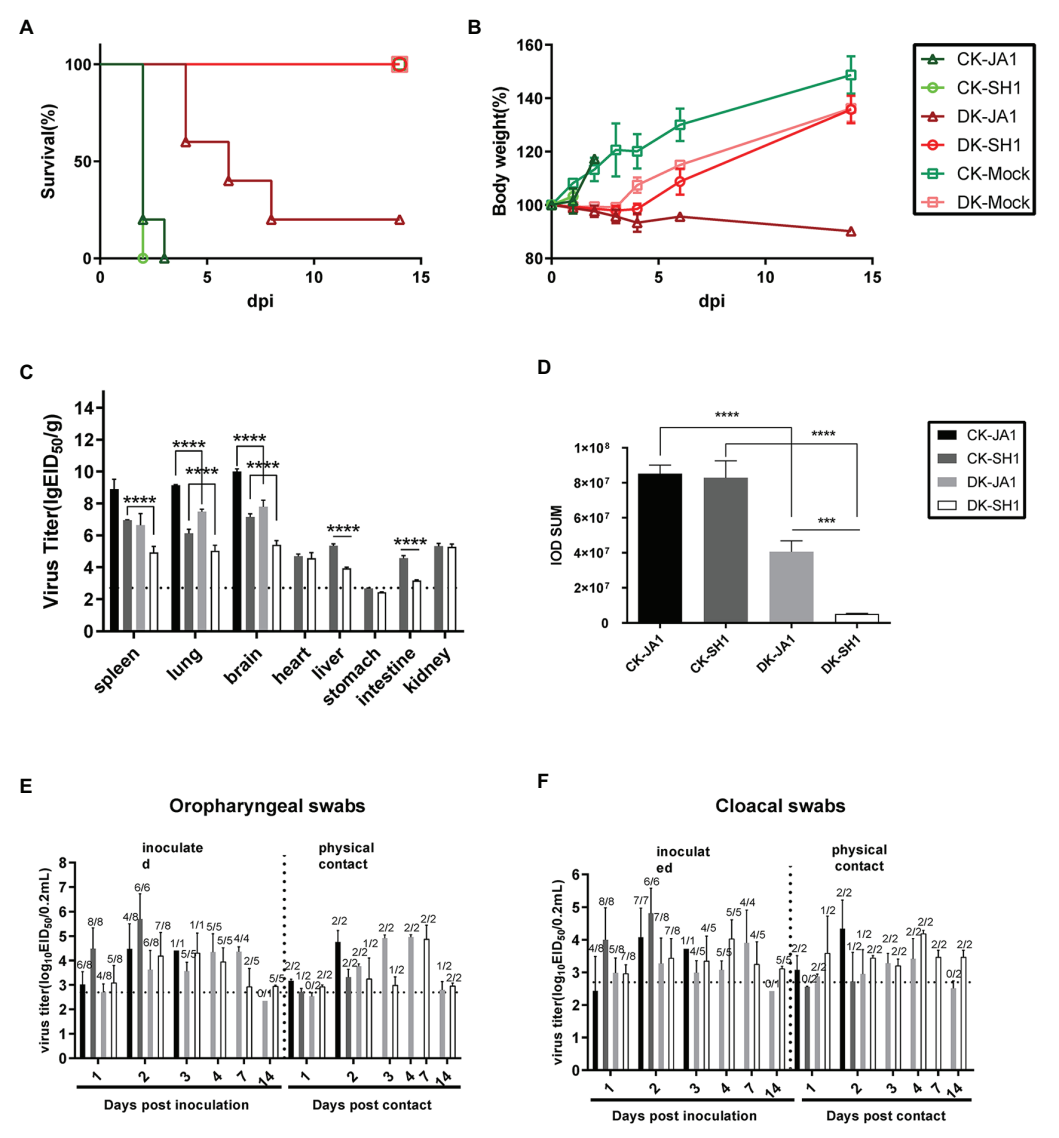
Pathogenicity and transmissibility of the two H5N6 viruses in chickens and ducks. **(A,B)** Survival and weight changes of H5N6 infected chickens and ducks. **(C)** Comparison of two A(H5N6) influenza virus titers in tissues of chickens and ducks. Virus titers were determined by quantitative real-time polymerase chain reaction (qRT-PCR) and expressed as log_10_ EID_50_/g of tissue. Data are expressed as mean values ± standard deviation. Black dashed lines indicate the lowest limit of detection. Differences were analyzed with Student’s *t*-test (^*^p < 0.05, ^**^p < 0.01, ^***^p < 0.001, ^****^p < 0.0001). **(D)** Representative quantification of viral NP staining in lung of H5N6-infected chickens and ducks. Staining was expressed as integral optical density (IOD), using the image pro plus 6.0. Viral loads of JA1 and SH1 in oropharyngeal swabs **(E)** and cloacal swabs **(F)** in H5N6 influenza virus-infected and physical contact chickens and ducks. Virus loads were detected by qRT-PCR and are expressed as log_10_ EID_50_/ml. Results are expressed as mean values ± standard deviation. The proportion of positive bird swabs from all detected swabs is shown in the figure above each bar. Black dashed lines indicate the lowest limit of detection. CK-JA1, JA1 infected chickens; CK-SH1, SH1 infected chickens; DK-JA1; JA1 infected ducks; DK-SH1, SH1 infected ducks; CK-Mock, PBS inoculated chickens; DK-Mock, PBS inoculated ducks.

To evaluate the pathogenicity of the two viruses in chickens and ducks, we intranasally inoculated SPF chickens and mallard ducks with 10^6.5^ EID50 of either JA1 or SH1 H5N6 viruses. All inoculated chickens developed clinical symptoms, including lethargy, anorexia, and hunched posture and died within 3 DPI (Figure 2A), with a mean death time (MDT) of 2–2.2 days (Table 2). In contrast, inoculated ducks exhibited none of these clinical signs initially, but between 4 and 8 DPI (Figure 2A), four of the five JA1-infected ducks died as a result of severe disease including weight loss (Figure 2B), neurological signs, ataxia, and cloudy eyes. SH1-innoculated ducks showed no signs of infection except for one duck with cloudy eyes.

**Table 2:** Mortality, virus shedding, and seroconversion of bird response to H5N6 virus infection.

Birds	Group	Infection^a^ (%)	Mortality (%)	MDT (d)	Oral shedding (log_10_EID_50_)	Cloacal shedding (log_10_EID_50_)	HI titer^b^ (log2)
Chickens	JA1	5/5(100)	5/5(100)	2.2	3.02–4.47	2.43–4.07	-^c^
	Contact	3/3(100)	3/3(100)	2	3.17–4.76	3.07–4.34	-^c^
	SH1	3/5(100)	3/5(100)	2	4.49–5.70	4.00–4.81	-^c^
	Contact	3/3(100)	3/3(100)	2	2.71–3.31	2.56–2.73	-^c^
Ducks	JA1	4/5(80)	4/5(80)	5.5	2.34–4.36	2.42–3.90	1/1(6)
	Contact	0/3(0)	0/3(0)	-^d^	2.54-4.96	2.51-3.42	3/3(5.67)
	SH1	1/5(20)	0/5(0)	-^d^	2.93-4.31	2.96-4.03	5/5(5.34)
	Contact	0/3(0)	0/3(0)	-^d^	2.93-4.88	3.20-4.19	3/3(5.33)

a Data show the ratio of infected ducks to the number of total birds and birds were considered virus positive if RNA was detected at 2 DPI.

b Data show the ratio of antibody-positive birds to the number of virus-inoculated birds and HI titer.

c All the chickens died at the end of the observation.

d These ducks did not die during the observation time.

HI, hemagglutination inhibition; MDT, mean death time.

Viruses were detected in many organs of the infected chickens and ducks at 2 DPI including the spleen, lung, brain, heart, liver, intestine, and kidney tissues (Figure 2C). The two viruses replicated in the spleen, lung, and brain of chickens, with mean titers of 6.95–8.90 log_10_ EID_50_, 6.13–9.15 log_10_ EID_50_, and 7.15–10.01 log_10_ EID_50_, respectively. In contrast, viral load was 4.93–6.64 log_10_ EID_50_, 5.03–7.49 log_10_ EID_50_, and 5.41–7.79 log_10_ EID_50_ in the spleen, lung, and brain of ducks. Higher viral loads were in general detected in tissues of chickens, including spleen, lung, brain, liver, and intestines, than in those of ducks (Figure 2C).

**Figure 3:**
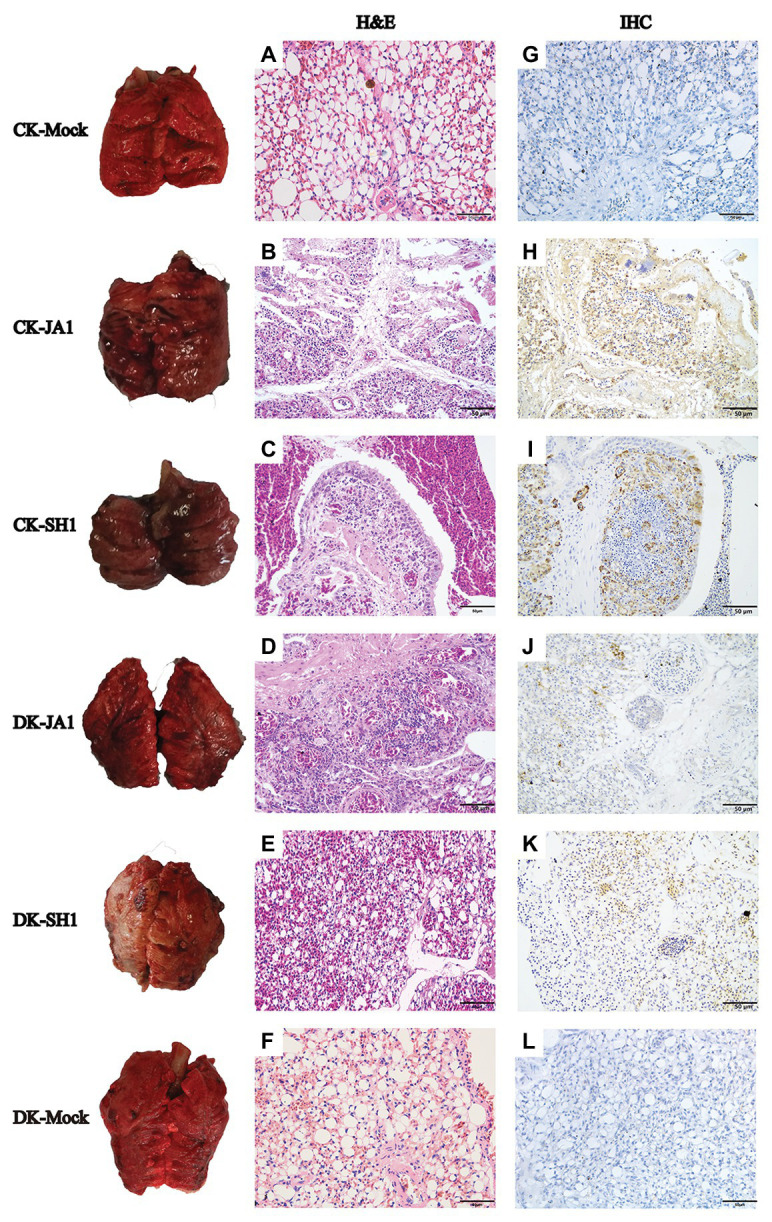
Gross and histopathology of lungs in chickens and ducks infected with H5N6 viruses. Representative gross lung (left column) and corresponding histological [H&E staining; middle column **(A–F)**] and immunohistochemical [right column **(G–L)**] lung sections are shown. Control groups of chickens and ducks inoculated with PBS **(A,G)**; JA1 and SH1 infections in chickens caused severe pneumonia with hemorrhage, inflammatory cell infiltrates, and pulmonary edema **(B,C)**; JA1 and SH1 infections in ducks, respectively, produced obvious and mild pneumonia **(D,E)**. Extensive localization of viral NP to pulmonary capillary, lymphocytes, and vascular endothelial cells for both viruses **(H–K)**.

The pathological findings of H5N6 influenza virus infection in ducks, evaluated by a pathologist in a blinded manner, were consistent with the comparatively mild clinical response, but chickens showed severe lung damage (Figure 3). Microscopic lesions from H5N6 virus-infected lungs of chickens showed severe lesions, with severe and extensive pulmonary edema, interlobular septal thickening, congestion and/or hemorrhage, massive inflammatory cell infiltration into pulmonary capillary (Figures 3B,C). JA1 virus-infected lungs of ducks showed obvious pneumonia (Figure 3D). SH1 virus-infected lungs of ducks had mild pneumonia (Figure 3E). Viral NP of H5N6 viruses could be easily detected in the pulmonary capillary epithelial cell, lymphocytes, and vascular endothelial cells of infected chickens and ducks (Figures 3H–K). Immunohistochemical staining of NP showed stronger staining in lung tissue of chickens in comparison to lung tissue of ducks according to a quantitative evaluation (Figure 2D).

### Transmissibility of the Two H5N6 Viruses in Chickens and Ducks

To assess the horizontal intraspecies transmissibility of the two H5N6 viruses in chickens and ducks, four birds were intranasally inoculated with 0.2 ml PBS as a naïve contact group, which were then co-housed with birds inoculated with JA1 or SH1 (Supplementary Tables S2, S3). They exhibited 100% mortality, with MDT of 2.0–2.2 days (Table 2). Shedding of JA1 could be detected from both oropharyngeal and cloacal swabs of virus-inoculated and contact group chickens within 2 DPI, with viral load in the ranges of 3.02–4.76 log_10_ EID_50_ in oropharyngeal swab samples and of 2.43–4.34 log_10_ EID_50_ in cloacal swab samples. SH1 inoculated chickens shed virus at high-viral loads orally and cloacally (4.00–5.70 log_10_ EID_50_), but most naive contact chickens did not shed virus (Figures 2E,F; Table 2).

In contrast, naïve contact ducks co-housed with H5N6-infected ducks did not die during the observation time. JA1 could be detected from the oropharynx and cloaca of virus-inoculated and contact group ducks within 7 DPI, with mean viral loads of 2.34–4.96 log_10_ EID_50_ and of 2.42–3.90 log_10_ EID_50_, respectively (Figures 2E,F; Table 2). Shedding of SH1 was observed in virus-inoculated and contact group ducks within 14 DPI, with viral loads of 2.93–4.88 log_10_ EID_50_ in oropharyngeal swabs and 2.96–4.19 log_10_ EID_50_ in cloacal swabs (Figures 2E,F). All surviving ducks seroconverted and exhibited high titers (5.33–6 log2; Table 2).

### Expression of PRRs, Cytokines, and Inflammatory Mediators in the Target Tissues of the H5N6 Virus-Infected Chickens and Ducks

**Figure 4:**
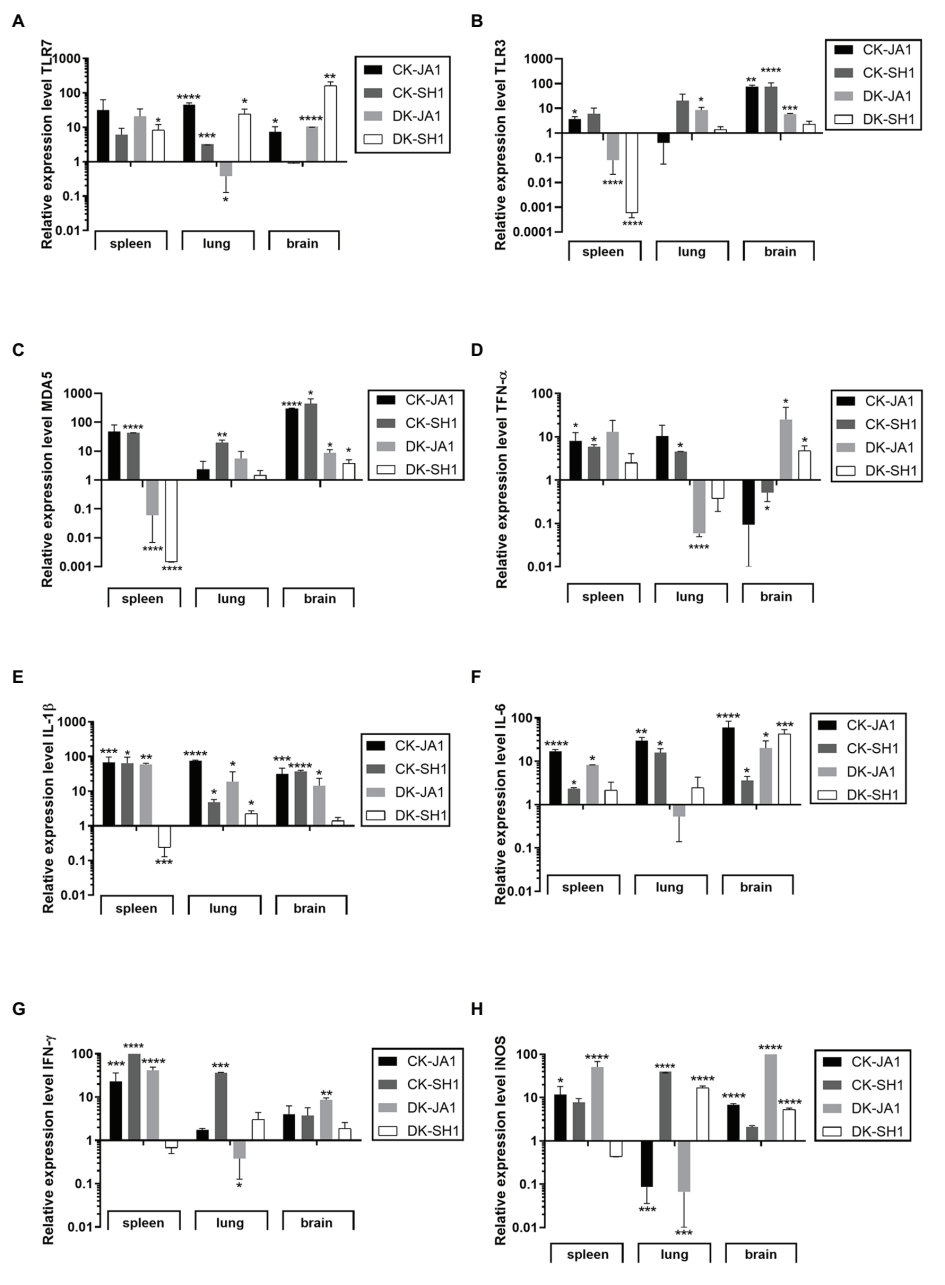
Expression profiles of TLRs, proinflammatory cytokines, and inflammation mediators in the target tissues of chickens and ducks infected with H5N6. At 2 days post-infection, the target tissues (i.e., spleen, lung, and brain) of H5N6-infected chickens and ducks were harvested for detection of immune gene at mRNA level *via* qRT-PCR. **(A)** TLR7, **(B)** TLR3, **(C)** MDA5, **(D)** TNF-α, **(E)** IL-1β, **(F)** IL-6, **(G)** IFN-γ, and **(H)** iNOS. Each bar represents the level of target gene mRNA relative to mock after normalizing to GAPDH. Data are expressed as the mean values ± standard deviation. Statistical analysis was performed with Student’s *t*-test (^*^p < 0.05, ^**^p < 0.01, ^***^p < 0.001, ^****^p < 0.0001). TLRs, Toll-like receptors; qRT-PCR, quantitative real-time polymerase chain reaction; and GAPDH, glyceraldehyde 3-phosphate dehydrogenase.

TLRs are important types of PRRs in immune systems that recognize influenza virus (Hamerman et al., 2016). In the study, we detected the mRNA levels of TLR7, TLR3, and MDA5 in the spleen, lung, and brain at 2 DPI to assess the expression of PRRs in H5N6 virus-infected chickens and ducks. As shown in Figures 4A–C, the expression levels of TLR7, TLR3, and MDA5 in the spleen of chickens were upregulated when induced by JA1 (31.46-fold, 3.70-fold, and 48.15-fold) and SH1 (6.11-fold, 6.05-fold, and 43.13-fold) viruses compared to uninfected controls. In the lung of chicken, TLR 7 expression level was upregulated significantly by JA1 with a fold of 45.97, and MDA5 expression level was induced remarkably by SH1 with 19.89-fold. Engagement of TLRs leads to production of inflammatory cytokines, including TNF-α, IL-1β, IL-6, and IFN-γ (Medzhitov, 2001). The expression of TNF-α was increased in the spleen and lung after infection with JA1 and SH1 with a fold change of 4.51–10.39, yet decreased in the brain with a fold change of 0.09–0.52 (Figure 4D). In contrast to uninfected chickens, the expression levels of IL-1β and IL-6 were significantly upregulated in the spleen, lung, and brain when triggered by JA1 (17.03–75.16-fold) and SH1 (2.36–64.69-fold; Figures 4E,F). JA1 and SH1 viruses significantly induced the expression of IFN-γ in spleen with a fold increase of 23.17–98.91 (Figure 4G). The iNOS is an indicator of inflammatory mediator NO (Ying et al., 2013). Expression profiling of iNOS genes in the tested tissues of infected chickens and ducks reveals a complex pattern (Figure 4H).

However, ducks responded differentially to different H5N6 strains. When induced by JA1, TLR7 expression was upregulated in the spleen by 20.88-fold, and TLR3 expression was upregulated in the lung by 3.84-fold (Figures 4A,B). TLR7, TLR3, and MDA5 mRNA of JA1 virus-infected ducks showed higher levels of activation in the brain compared to the control (10.59-fold, 5.91-fold, and 8.83-fold, *p* < 0.0001, *p* = 0.0002, and *p* = 0.0315, respectively; Figures 4A–C). In the spleen and brain of ducks, JA1 induced significantly upregulated expression levels of TNF-α, IL-6, IFN-γ, and iNOS by 13.05-fold and 25.27-fold, 8.25-fold and 20.12-fold, 41.65-fold and 8.59-fold, and 62.91-fold and 146.40-fold, respectively (*p* < 0.05) compared to uninoculated ducks, but downregulated in the lung (0.06-fold, 0.53-fold, 0.38-fold, and 0.07-fold, respectively; Figures 4D,F–H). And the expression of IL-1β was increased in response to JA1 virus infection in the spleen, lung, and brain by 59.72-fold, 6.86-fold, and 14.42-fold (Figure 4E). While in the SH1-infected ducks, TLR7 mRNA in spleen, lung, and brain exhibited significant activation (8.34–164.11-fold, *p* < 0.05), and MDA5 and RIG-I mRNA in brain were significantly upregulated (3.85-fold and 3.99-fold, *p* < 0.0001 and *p* < 0.05, respectively) over that of control (Figures 4A–C; Supplementary Figure S2). Accordingly, the mRNA levels of TNF-α, IL-6, and iNOS were upregulated in the brain of SH1-inoculated ducks compared to uninfected ducks with 4.77-fold, 43.25-fold, and 146.40-fold (*p* < 0.05; Figure 4B). But, the expression level of IL-1β, IFN-γ, and iNOS in the spleen were downregulated by 0.24-fold, 0.67-fold, and 0.44-fold compared to that of control ducks.

**Figure 5:**
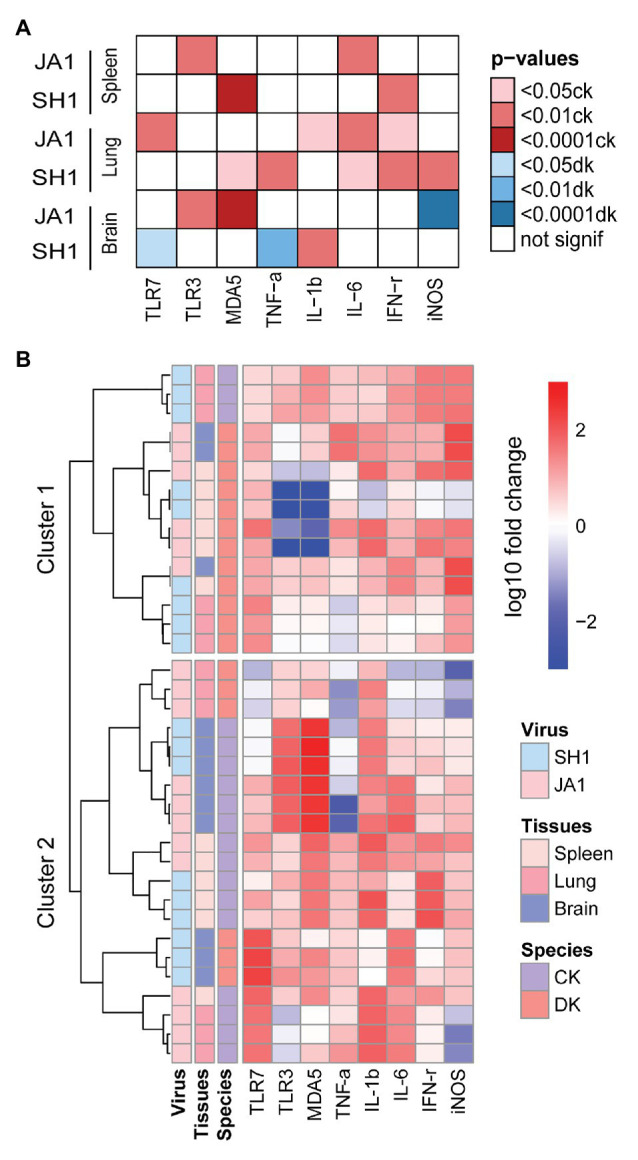
Heatmap of gene expression responsive to influenza A (H5N6) virus in the spleens, lungs and brains of chickens and ducks on day 2 after inoculation. **(A)** Significance of specie in relation to different cytokines (x axis) induced by two H5N6 strains (y axis). Only the resulting significant differences are shown in this plot. The darker the color, the greater the significance, red indicates a stronger response in chickens and blue indicates a stronger response in ducks (see figure legend). **(B)** Unbiased heat map comparisons of cytokines in tissues. Measurements were normalized across all avian. Hierarchical clustering was used to determine clusters 1–2 (cluster 1, *n* = 15 and cluster 2, *n* = 21).

Overall, H5N6 HPAIVs induced gene expression variability in chickens and ducks (Figure 5A; Supplementary Tables S6–S8). When infected by SH1, significant higher TNF-α, IL-6, IFN-γ, and iNOS were detected in the lung of chickens compared to ducks. In addition, chickens show higher IFN-γ in the spleen and increased IL-6 in the brain than ducks. While in chickens infected by JA1, we find significant higher expression levels of IL-1β, IL-6, and IFN-γ in the lung and increased production of IL-6 in the spleen compared to those in ducks. With regard to virus strains, JA1 virus induced a higher expression of IL-1β, IL-6, and IFN-γ in the spleen of ducks than SH1 virus (Supplementary Table S9). In the brain, ducks infected with JA1 virus exhibited significant higher expression of iNOS than those infected with SH1 (Supplementary Table S10).

To further evaluate potential drivers of severe H5N6 HPAIVs outcome in an unbiased manner, we performed unsupervised clustering analysis that included two species, two H5N6 strains, and three tissue types using PRRs, cytokines, and inflammatory mediators (Figure 5B). Notably, two main clusters emerged: cluster 1 primarily comprised ducks that showed reduced mortality and was characterized by low levels of inflammatory markers and clusters 2 primarily contained chickens with 100% mortality and was driven by a set of inflammatory markers. In summary, our results revealed that the mRNA expression profiles of innate immune gene displayed different patterns in chickens and ducks.

## Discussion

In this study, we found that both two H5N6 HPAIVs (JA1 and SH1) were highly pathogenic in chickens. All infected chickens died and shed virus. While in mallard ducks, JA1 had high pathogenicity, but SH1 was non-pathogenic. SH1-inoculated ducks shed virus for longer times than JA1-inoculated ducks, indicating that SH1 replicated better in ducks (Pantin-Jackwood et al., 2017). Both viruses showed efficient horizontal interspecies transmissibility in chickens and ducks. All contact chickens died but none of contact ducks did. Most contact chickens of SH1 inoculated group did not shed detectable virus, whereas all contact ducks shed virus, suggesting that SH1 was transmissible in ducks, but transmitted less efficiently in chickens and SH1 have adapted to ducks rather than to chickens (Zhu et al., 2013; Shi et al., 2016). Long-term shedding is of great consequence in asymptomatic ducks in that it increases the possibility of transmission of the virus, posing a threat to poultry (Hulse-Post et al., 2005).

We have further compared putative amino acid sequences between the two H5N6 HPAIVs to reveal any relationship between the amino acid sequences of the viruses and pathogenicity in chickens and ducks. JA1 and SH1 H5N6 viruses possessed multiple basic amino acid residues (RERRRKR↓GLF) at cleavage site of HA, which suggested that both of them are highly pathogenic for chickens (Liu et al., 2005; Li et al., 2017b). The combination of Q226/G228 in the two viruses favored avian-like SAα2,3Gal recognition and conferred increased expression of proinflammatory response (Ramos et al., 2011). Both an 11-aa deletion in NA and a 5-aa deletion in NS were found in the two viruses, which enhanced replication and virulence in chickens (Long et al., 2008; Munier et al., 2010). These mutations may contribute to the high pathogenicity of H5N6 in chickens. Previous analysis indicated that four C-terminal residues of the NS1 protein is a PL of the X-S/T-X-V type. Substitution of the NS1 C terminus with ESEV significantly increased the virulence and pathogenicity of the virus in infected mice (Obenauer et al., 2006; Jackson et al., 2008). Genetic analysis showed that NS1 protein from JA1 contained the predominant avian PL motif ESEV, and the NS1 protein from SH1 contained the motif ESEI, which might be a key contributor for the different pathogenicity of the H5N6 virus in ducks. In addition, SH1 expresses full-length PB1-F2 (90aa), but JA1 has a N-terminally truncated PB1-F2 (52aa), which is a variant of unknown significance. Full length PB1-F2 proteins from H5N1 and pandemic strains of the 20ths enhance immunopathology (McAuley et al., 2007; Julie et al., 2010), but truncated PB1-F2 protein of 11 amino acids at the C terminus increases the virulence of the H7N9 virus in mice (Su et al., 2015). JA1 has the amino acid residues QQG at positions 222–224 in HA receptor binding region, although the corresponding amino acid sequences of JA1 are QRG. These molecular characteristics may provide clues to different pathogenicity and transmissibility of two strains of H5N6 viruses in ducks. Phenotypic relevance of mutations could be characterized further by introduction of mutation and by using reverse genetics.

Previous studies have revealed that cytokine storms, defined by the aberrant and exaggerated production of pro-inflammatory cytokines, are associated with the morbidity and mortality of H1N1 and H5N1 influenza virus in mouse (Cilloniz et al., 2010), macaques (Kobasa et al., 2007; Cillóniz et al., 2009), and humans (de Jong et al., 2006; Fajgenbaum and June, 2020). Phagocytosis of influenza virus by macrophages can stimulate the secretion of antiviral (IFN-γ) and inflammatory (TNF-α, IL-1β, and IL-6) cytokines and oxidative and nitrosative stress enzymes (iNOS), increasing vascular permeability and lymphocyte recruitment locally and inducing the release of acute phase proteins systemically (Marois et al., 2012). The inflammatory response is beneficial when cytokines are released at appropriate levels, but harmful when produced in a deregulated fashion (Arango Duque and Descoteaux, 2014; Liu et al., 2020).

Notably, chickens infected H5N6 showed severe pneumonia (lethargy and anorexia), with MDT of 2–2.2 days, but ducks infected with JA1 showed neurological clinical signs (tremors, circling, and loss of balance), with MDT of 5.5 days, and ducks infected with SH1 showed no obvious clinical signs or death. Consistently, H5N6 viruses caused higher viral loads in all tested tissues in chickens when compared to ducks. In addition, immune response associated with H5N6 infections of chickens and ducks are different (Figures 4, 5). Our study showed that chickens infected with JA1 had increased production of IL-6 in comparison to ducks. This increase appeared to be systemic since elevated levels were found in the spleen, lung, and brain, consistent with previous study on H5N1 (Burggraaf et al., 2014). Aberrant IL-6 is involved in the regulation of sickness behavior (e.g., anorexia and lethargy) and directly linked to host morbidity (Walsh et al., 2011). We also observed that expression levels of TNF-α, IFN-γ, and iNOS were significantly higher in the lungs of chickens infected with SH1 than those in ducks. Combination of TNF-α and IFN-γ triggers inflammatory cell death, tissue damage, and mortality, by inducing nitric oxide production and driving caspase8/FADD-mediated PANoptosis in the lung, which leads to the release of cellular contents including inflammatory cytokines and alarmins to propagate and exacerbate the systemic response defined as cytokine storm (Walsh et al., 2011; Karki et al., 2020). The differential responses of chicken and ducks to H5N6 might further be explained by species-specific arrangements of PRR such as the higher TLR7 and MDA5 expression levels in the lung of chickens. Our results revealed that these two H5N6 HPAIVs induced higher pathogenic and inflammatory effects in chickens compared to ducks.

Newly evolved H5N1 HPAIVs have switched their tissue tropism to be more neurotropic in ducks naturally, which has been linked to death of infected ducks (Sturm-Ramirez et al., 2004; Samir et al., 2020). Our study showed the expression of iNOS in response to JA1 was remarkably higher than for SH1 in the brain, which may contribute to severe neurological signs and death (Burggraaf et al., 2011; Hosseini et al., 2018). iNOS produces an excessive amount of NO for a longer time, which allows generation of peroxynitrite *via* a radical coupling reaction of NO with superoxide anion. Thus, peroxynitrite causes overwhelming oxidative tissue injury through potent oxidation and nitration reactions, committing cells to necrosis or apoptosis (Pacher et al., 2007). NO also appears to regulate a host’s immune response, with immunopathological consequences, and has pathological effects in influenza virus infections (Akaike and Maeda, 2000). Increased iNOS expression may be associated with higher severity of H5N6 influenza virus infection, which was consistent with the previous study on H5N1 (Burggraaf et al., 2011). iNOS were downregulated in lung tissues of JA1-infected ducks, indicating that the cytokines are expressed in a tissue-specific manner. We also observed the upregulation of IL-1β, IL-6, IFN-γ, and iNOS in the spleens of JA1-infected ducks. But, IL-1β, IFN-γ, and iNOS were downregulated in the spleen of ducks infected with SH1, and IL-6 was slightly upregulated, consistent with limited clinical signs. Recent study showed the H5N6 viruses that had higher pathogenicity in mice induced stronger inflammatory responses (Xiang et al., 2020). These results are consistent with previous studies of H5N1 and H5N6 infection in chickens, ducks, and mice, which showed that higher pathogenic viruses induced stronger inflammatory responses (Wei et al., 2013; Gao et al., 2017; Xiang et al., 2020).

In the study, we observed the significant higher pathogenic and inflammatory effect in chickens than ducks, but the relevance between cytokine profile and pathology of H5N6 virus should be verified by comprehensive analysis of diverse inflammatory dynamics in a greater number of animals infected with different H5N6 isolates. We identified higher inflammatory response in chickens during the early phase (incubation period; <2 DPI; World Health Organization Writing Group et al., 2006), but late-stage pathology in ducks may be driven primarily by host immune responses during late phase (prodromal or illness period), which required detailed prospective study to confirm this speculation. The previous study indicated that, after infection with the highly pathogenic H5N1 and H7N1 avian influenza viruses, natural hosts for influenza virus, such as wild ducks, usually develop a limited inflammation of the respiratory system, with little or no mortality. By contrast, in spillover hosts such as chickens and humans, infection with HPAIVs can induce cytokine storm in the respiratory system and the infection can cause severe disease and high mortality (Lee et al., 2007; Karpala et al., 2011; Bean et al., 2013; Cornelissen et al., 2013; Chan et al., 2015). Observations in our subjects support that H5N6 HPAIVs induce higher pathogenic and inflammatory effects in chickens than ducks. The mechanism that induces a limited inflammatory response in ducks remains unknown. Further study is required to identify all the contributing factors. Uncovering the differences between the natural hosts and spillover hosts might enable targeted therapeutic intervention (Morse et al., 2012; Bean et al., 2013).

In sum, both H5N6 AIVs were highly pathogenic and efficiently transmitted in chickens, for higher virus titers appeared in tested tissues early in the infection period, and were accompanied by higher inflammatory response. Importantly, the TNF-α, IL-6, IFN-γ, and iNOS levels were significantly higher in the lung of SH1-infected chickens compared to those of ducks. And we find higher systemic levels of IL-6 induced by JA1 in chickens than in ducks. In addition, JA1 caused higher mortality and higher iNOS level in the brain accompanied by neurological signs in ducks. These results are helpful to understand the relationship between the pathogenicity of H5N6 AIVs and inflammatory responses to them in chickens and ducks.

## Data Availability Statement

The datasets presented in this study can be found in online repositories. The names of the repository/repositories and accession number(s) can be found below: https://www.ncbi.nlm.nih.gov/genbank/, MH022713, MH022714, MH022715, MH022716, MH022717, MH022718, MH022719, MH022720.

## Ethics Statement

The animal study was reviewed and approved by Committee on the Ethics of Animal Experiments in the Institute of Zoology, Chinese Academy of Sciences.

## Author Contributions

HH took the lead in designing and performing the study. BW performed the experiments, processed the experimental data and analysis, drafted the manuscript, and designed the figures. JL and ML assisted on virus isolation and genome sequences. SH, GY, YH, and MG aided in the animal experiments. QS and CH were involved in the experiments in molecular biology. QS, ML, and JM helped to carry out the genetic analysis. QW performed the pathologic analysis. QS, JL, JD, HC, and QZ contributed to write and revise the manuscript. All authors contributed to the article and approved the submitted version.

### Conflict of Interest

The authors declare that the research was conducted in the absence of any commercial or financial relationships that could be construed as a potential conflict of interest.
