# Supplementary material for: Differences in Highly Pathogenic H5N6 Avian Influenza Viral Pathogenicity and Inflammatory Response in Chickens and Ducks
Source: Front Microbiol. 2021 Jan 29;12:593202. doi: 10.3389/fmicb.2021.593202 (PMC7878534; doi:10.3389/fmicb.2021.593202)
Supplement: Supplementary file 1 [file Presentation_1.pdf]

Supplementary Material

Pathogenicity of highly pathogenic H5N6 avian influenza viruses and associated inflammatory response in chickens and ducks

Bo Wang[1,2], Qianqian Su[1,2], Jing Luo[1], Meng Li[1], Qiaoxing Wu[1], Chengmei Huang[1,2], Juan Du[1,2], Jiajun Ma[1,2], Han Chang[1,2], Shuyi Han[1], Guohui Yuan[1], Yapeng He[1], Minglei Guo[1], Qingxun Zhang[1], Hongxuan He[1]*

[1] National Research Center for Wildlife-Borne Diseases, Institute of Zoology, Chinese Academy of Sciences, Beijing, China

[2] College of Life Science, University of Chinese Academy of Sciences, Beijing, China

*** Correspondence:**

Corresponding Author: Hongxuan He, Ph.D., Executive Deputy Director of National Research Centre for Wildlife Borne Diseases, Institute of Zoology, Chinese Academy of Sciences. Add: No.1-5 Beichenxilu, Chaoyang District, Beijing 100101, P.R. China. Tel: 86-10-64807118(O/F). E-mail: hehx@ioz.ac.cn

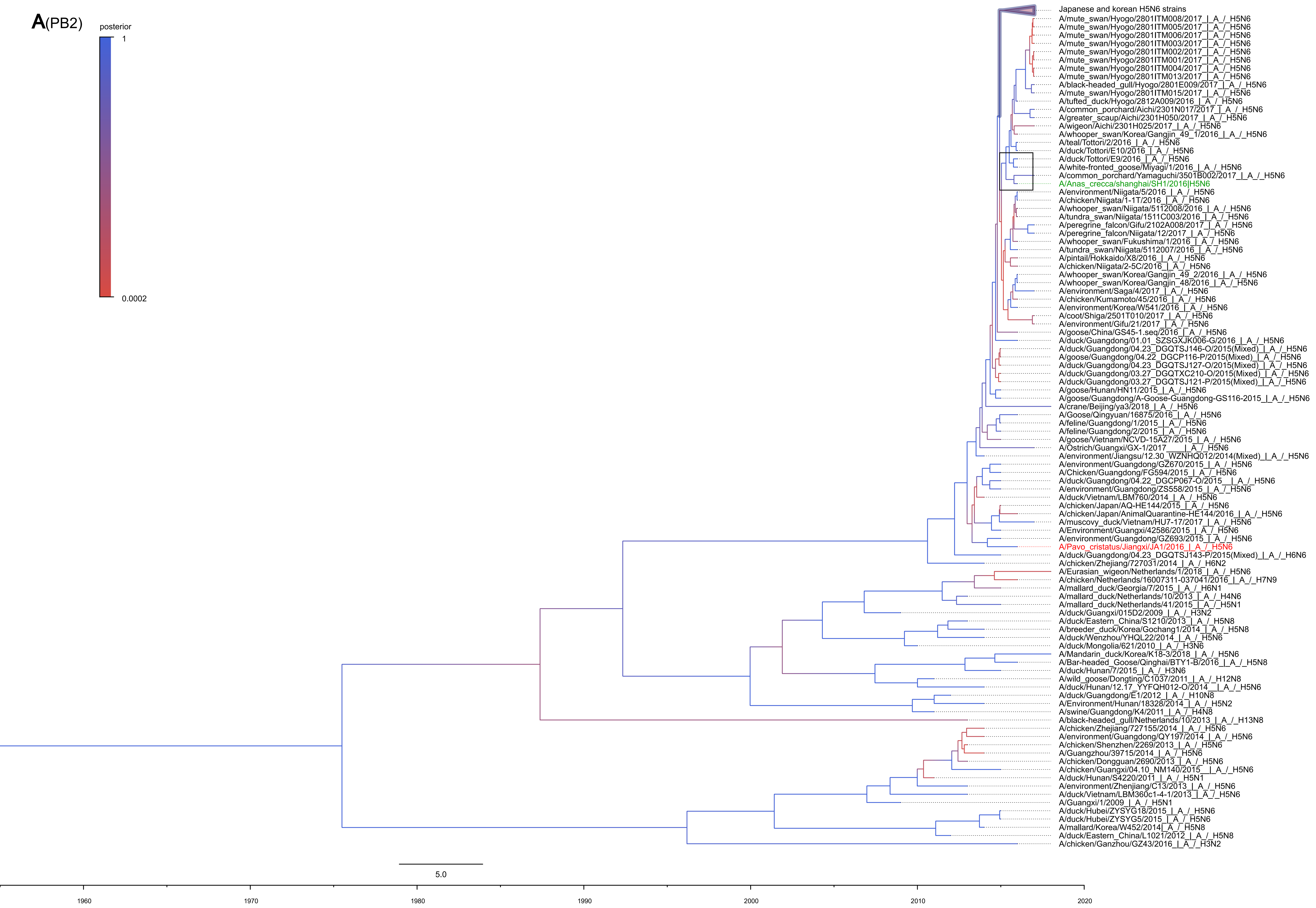

A(PB2)
posterior
1
0.0002
Japanese and korean H5N6 strains
A/mute_swan/Hyogo/2801ITM008/2017_|_A_/_H5N6
A/mute_swan/Hyogo/2801ITM005/2017_|_A_/_H5N6
A/mute_swan/Hyogo/2801ITM006/2017_|_A_/_H5N6
A/mute_swan/Hyogo/2801ITM003/2017_|_A_/_H5N6
A/mute_swan/Hyogo/2801ITM002/2017_|_A_/_H5N6
A/mute_swan/Hyogo/2801ITM001/2017_|_A_/_H5N6
A/mute_swan/Hyogo/2801ITM004/2017_|_A_/_H5N6
A/mute_swan/Hyogo/2801ITM013/2017_|_A_/_H5N6
A/black-headed_gull/Hyogo/2801E009/2017_|_A_/_H5N6
A/mute_swan/Hyogo/2801ITM015/2017_|_A_/_H5N6
A/tufted_duck/Hyogo/2812A009/2016_|_A_/_H5N6
A/common_porchard/Aichi/2301N017/2017_|_A_/_H5N6
A/greater_scaup/Aichi/2301H050/2017_|_A_/_H5N6
A/wigeon/Aichi/2301H025/2017_|_A_/_H5N6
A/whooper_swan/Korea/Gangjin_49_1/2016_|_A_/_H5N6
A/teal/Tottori/2/2016_|_A_/_H5N6
A/duck/Tottori/E10/2016_|_A_/_H5N6
A/duck/Tottori/E9/2016_|_A_/_H5N6
A/white-fronted_goose/Miyagi/1/2016_|_A_/_H5N6
A/common_porchard/Yamaguchi/3501B002/2017_|_A_/_H5N6
A/Anas_crecca/shanghai/SH1/2016|H5N6
A/environment/Niigata/5/2016_|_A_/_H5N6
A/chicken/Niigata/1-1T/2016_|_A_/_H5N6
A/whooper_swan/Niigata/5112008/2016_|_A_/_H5N6
A/tundra_swan/Niigata/1511C003/2016_|_A_/_H5N6
A/peregrine_falcon/Gifu/2102A008/2017_|_A_/_H5N6
A/peregrine_falcon/Niigata/12/2017_|_A_/_H5N6
A/whooper_swan/Fukushima/1/2016_|_A_/_H5N6
A/tundra_swan/Niigata/5112007/2016_|_A_/_H5N6
A/pintail/Hokkaido/X8/2016_|_A_/_H5N6
A/chicken/Niigata/2-5C/2016_|_A_/_H5N6
A/whooper_swan/Korea/Gangjin_49_2/2016_|_A_/_H5N6
A/whooper_swan/Korea/Gangjin_48/2016_|_A_/_H5N6
A/environment/Saga/4/2017_|_A_/_H5N6
A/chicken/Kumamoto/45/2016_|_A_/_H5N6
A/environment/Korea/W541/2016_|_A_/_H5N6
A/coot/Shiga/2501T010/2017_|_A_/_H5N6
A/environment/Gifu/21/2017_|_A_/_H5N6
A/goose/China/GS45-1.seq/2016_|_A_/_H5N6
A/duck/Guangdong/01.01_SZSGXJK006-G/2016_|_A_/_H5N6
A/duck/Guangdong/04.23_DGQTSJ146-O/2015(Mixed)_|_A_/_H5N6
A/goose/Guangdong/04.22_DGCP116-P/2015(Mixed)_|_A_/_H5N6
A/duck/Guangdong/04.23_DGQTSJ127-O/2015(Mixed)_|_A_/_H5N6
A/duck/Guangdong/03.27_DGQTXC210-O/2015(Mixed)_|_A_/_H5N6
A/duck/Guangdong/03.27_DGQTSJ121-P/2015(Mixed)_|_A_/_H5N6
A/goose/Hunan/HN11/2015_|_A_/_H5N6
A/goose/Guangdong/A-Goose-Guangdong-GS116-2015_|_A_/_H5N6
A/crane/Beijing/ya3/2018_|_A_/_H5N6
A/Goose/Qingyuan/16875/2016_|_A_/_H5N6
A/feline/Guangdong/1/2015_|_A_/_H5N6
A/feline/Guangdong/2/2015_|_A_/_H5N6
A/goose/Vietnam/NCVD-15A27/2015_|_A_/_H5N6
A/Ostrich/Guangxi/GX-1/2017____|_A_/_H5N6
A/environment/Jiangsu/12.30_WZNHQ012/2014(Mixed)_|_A_/_H5N6
A/environment/Guangdong/GZ670/2015_|_A_/_H5N6
A/Chicken/Guangdong/FG594/2015_|_A_/_H5N6
A/duck/Guangdong/04.22_DGCP067-O/2015__|_A_/_H5N6
A/environment/Guangdong/ZS558/2015_|_A_/_H5N6
A/duck/Vietnam/LBM760/2014_|_A_/_H5N6
A/chicken/Japan/AQ-HE144/2015_|_A_/_H5N6
A/chicken/Japan/AnimalQuarantine-HE144/2016_|_A_/_H5N6
A/muscovy_duck/Vietnam/HU7-17/2017_|_A_/_H5N6
A/Environment/Guangxi/42586/2015_|_A_/_H5N6
A/environment/Guangdong/GZ693/2015_|_A_/_H5N6
A/Pavo_cristatus/Jiangxi/JA1/2016_|_A_/_H5N6
A/duck/Guangdong/04.23_DGQTSJ143-P/2015(Mixed)_|_A_/_H6N6
A/chicken/Zhejiang/727031/2014_|_A_/_H6N2
A/Eurasian_wigeon/Netherlands/1/2018_|_A_/_H5N6
A/chicken/Netherlands/16007311-037041/2016_|_A_/_H7N9
A/mallard_duck/Georgia/7/2015_|_A_/_H6N1
A/mallard_duck/Netherlands/10/2013_|_A_/_H4N6
A/mallard_duck/Netherlands/41/2015_|_A_/_H5N1
A/duck/Guangxi/015D2/2009_|_A_/_H3N2
A/duck/Eastern_China/S1210/2013_|_A_/_H5N8
A/breeder_duck/Korea/Gochang1/2014_|_A_/_H5N8
A/duck/Wenzhou/YHQL22/2014_|_A_/_H5N6
A/duck/Mongolia/621/2010_|_A_/_H3N6
A/Mandarin_duck/Korea/K18-3/2018_|_A_/_H5N6
A/Bar-headed_Goose/Qinghai/BTY1-B/2016_|_A_/_H5N8
A/duck/Hunan/7/2015_|_A_/_H3N6
A/wild_goose/Dongting/C1037/2011_|_A_/_H12N8
A/duck/Hunan/12.17_YYFQH012-O/2014__|_A_/_H5N6
A/duck/Guangdong/E1/2012_|_A_/_H10N8
A/Environment/Hunan/18328/2014_|_A_/_H5N2
A/swine/Guangdong/K4/2011_|_A_/_H4N8
A/black-headed_gull/Netherlands/10/2013_|_A_/_H13N8
A/chicken/Zhejiang/727155/2014_|_A_/_H5N6
A/environment/Guangdong/QY197/2014_|_A_/_H5N6
A/chicken/Shenzhen/2269/2013_|_A_/_H5N6
A/Guangzhou/39715/2014_|_A_/_H5N6
A/chicken/Dongguan/2690/2013_|_A_/_H5N6
A/chicken/Guangxi/04.10_NM140/2015__|_A_/_H5N6
A/duck/Hunan/S4220/2011_|_A_/_H5N1
A/environment/Zhenjiang/C13/2013_|_A_/_H5N6
A/duck/Vietnam/LBM360c1-4-1/2013_|_A_/_H5N6
A/Guangxi/1/2009_|_A_/_H5N1
A/duck/Hubei/ZYSYG18/2015_|_A_/_H5N6
A/duck/Hubei/ZYSYG5/2015_|_A_/_H5N6
A/mallard/Korea/W452/2014|_A_/_H5N8
A/duck/Eastern_China/L1021/2012_|_A_/_H5N8
A/chicken/Ganzhou/GZ43/2016_|_A_/_H3N2
5.0
1960
1970
1980
1990
2000
2010
2020

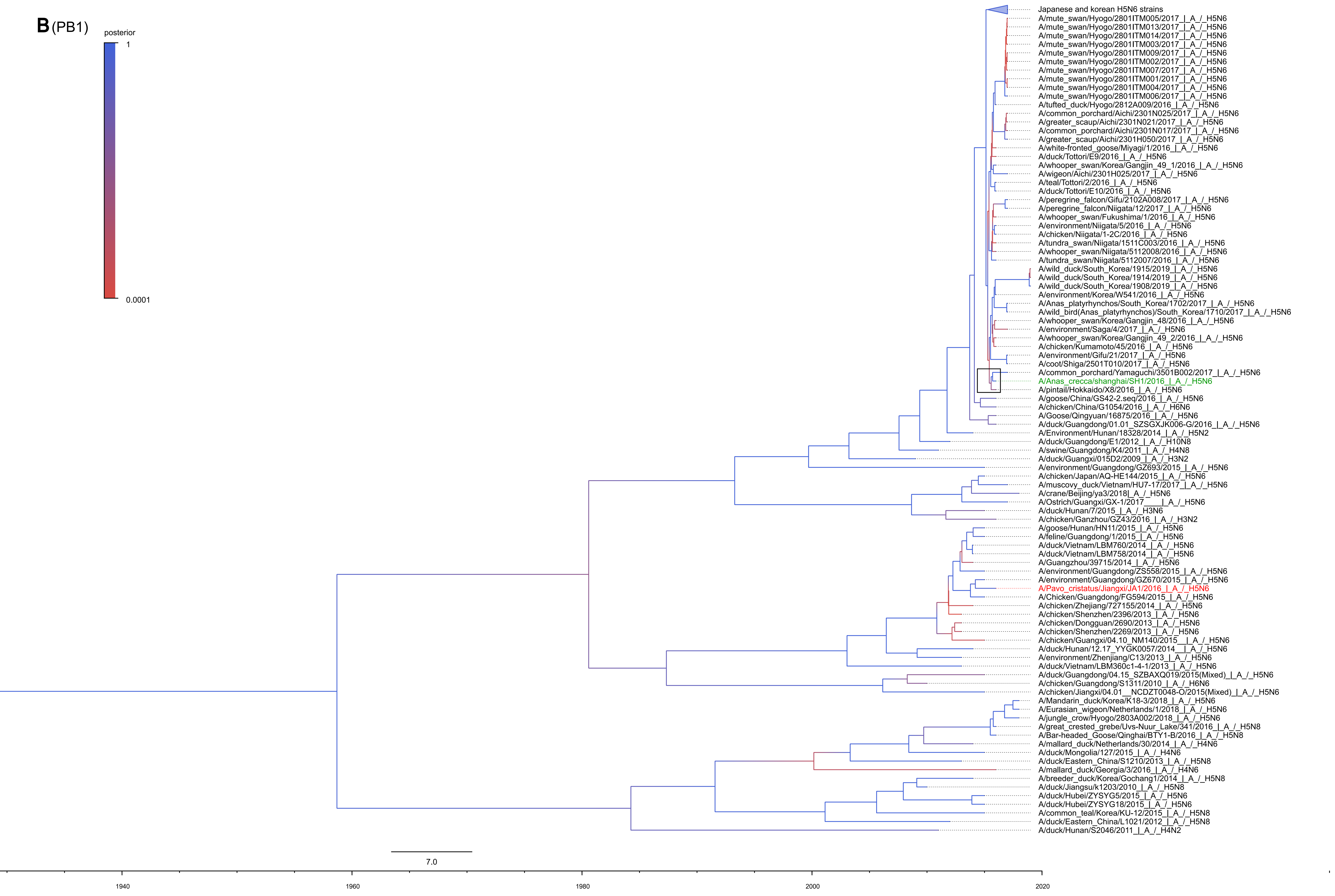
B (PB1)
posterior
1
0.0001
Japanese and korean H5N6 strains
A/mute_swan/Hyogo/2801ITM005/2017_|_A_/_H5N6
A/mute_swan/Hyogo/2801ITM013/2017_|_A_/_H5N6
A/mute_swan/Hyogo/2801ITM014/2017_|_A_/_H5N6
A/mute_swan/Hyogo/2801ITM003/2017_|_A_/_H5N6
A/mute_swan/Hyogo/2801ITM009/2017_|_A_/_H5N6
A/mute_swan/Hyogo/2801ITM002/2017_|_A_/_H5N6
A/mute_swan/Hyogo/2801ITM007/2017_|_A_/_H5N6
A/mute_swan/Hyogo/2801ITM001/2017_|_A_/_H5N6
A/mute_swan/Hyogo/2801ITM004/2017_|_A_/_H5N6
A/mute_swan/Hyogo/2801ITM006/2017_|_A_/_H5N6
A/tufted_duck/Hyogo/2812A009/2016_|_A_/_H5N6
A/common_porchard/Aichi/2301N025/2017_|_A_/_H5N6
A/greater_scaup/Aichi/2301N021/2017_|_A_/_H5N6
A/common_porchard/Aichi/2301N017/2017_|_A_/_H5N6
A/greater_scaup/Aichi/2301H050/2017_|_A_/_H5N6
A/white-fronted_goose/Miyagi/1/2016_|_A_/_H5N6
A/duck/Tottori/E9/2016_|_A_/_H5N6
A/whooper_swan/Korea/Gangjin_49_1/2016_|_A_/_H5N6
A/wigeon/Aichi/2301H025/2017_|_A_/_H5N6
A/teal/Tottori/2/2016_|_A_/_H5N6
A/duck/Tottori/E10/2016_|_A_/_H5N6
A/peregrine_falcon/Gifu/2102A008/2017_|_A_/_H5N6
A/peregrine_falcon/Niigata/12/2017_|_A_/_H5N6
A/whooper_swan/Fukushima/1/2016_|_A_/_H5N6
A/environment/Niigata/5/2016_|_A_/_H5N6
A/chicken/Niigata/1-2C/2016_|_A_/_H5N6
A/tundra_swan/Niigata/1511C003/2016_|_A_/_H5N6
A/whooper_swan/Niigata/5112008/2016_|_A_/_H5N6
A/tundra_swan/Niigata/5112007/2016_|_A_/_H5N6
A/wild_duck/South_Korea/1915/2019_|_A_/_H5N6
A/wild_duck/South_Korea/1914/2019_|_A_/_H5N6
A/wild_duck/South_Korea/1908/2019_|_A_/_H5N6
A/environment/Korea/W541/2016_|_A_/_H5N6
A/Anas_platyrhynchos/South_Korea/1702/2017_|_A_/_H5N6
A/wild_bird(Anas_platyrhynchos)/South_Korea/1710/2017_|_A_/_H5N6
A/whooper_swan/Korea/Gangjin_48/2016_|_A_/_H5N6
A/environment/Saga/4/2017_|_A_/_H5N6
A/whooper_swan/Korea/Gangjin_49_2/2016_|_A_/_H5N6
A/chicken/Kumamoto/45/2016_|_A_/_H5N6
A/environment/Gifu/21/2017_|_A_/_H5N6
A/coot/Shiga/2501T010/2017_|_A_/_H5N6
A/common_porchard/Yamaguchi/3501B002/2017_|_A_/_H5N6
A/Anas_crecca/shanghai/SH1/2016_|_A_/_H5N6
A/pintail/Hokkaido/X8/2016_|_A_/_H5N6
A/goose/China/GS42-2.seq/2016_|_A_/_H5N6
A/chicken/China/G1054/2016_|_A_/_H6N6
A/Goose/Qingyuan/16875/2016_|_A_/_H5N6
A/duck/Guangdong/01.01_SZSGXJK006-G/2016_|_A_/_H5N6
A/Environment/Hunan/18328/2014_|_A_/_H5N2
A/duck/Guangdong/E1/2012_|_A_/_H10N8
A/swine/Guangdong/K4/2011_|_A_/_H4N8
A/duck/Guangxi/015D2/2009_|_A_/_H3N2
A/environment/Guangdong/GZ693/2015_|_A_/_H5N6
A/chicken/Japan/AQ-HE144/2015_|_A_/_H5N6
A/muscovy_duck/Vietnam/HU7-17/2017_|_A_/_H5N6
A/crane/Beijing/ya3/2018|_A_/_H5N6
A/Ostrich/Guangxi/GX-1/2017___|_A_/_H5N6
A/duck/Hunan/7/2015_|_A_/_H3N6
A/chicken/Ganzhou/GZ43/2016_|_A_/_H3N2
A/goose/Hunan/HN11/2015_|_A_/_H5N6
A/feline/Guangdong/1/2015_|_A_/_H5N6
A/duck/Vietnam/LBM760/2014_|_A_/_H5N6
A/duck/Vietnam/LBM758/2014_|_A_/_H5N6
A/Guangzhou/39715/2014_|_A_/_H5N6
A/environment/Guangdong/ZS558/2015_|_A_/_H5N6
A/environment/Guangdong/GZ670/2015_|_A_/_H5N6
A/Pavo_cristatus/Jiangxi/JA1/2016_|_A_/_H5N6
A/Chicken/Guangdong/FG594/2015_|_A_/_H5N6
A/chicken/Zhejiang/727155/2014_|_A_/_H5N6
A/chicken/Shenzhen/2396/2013_|_A_/_H5N6
A/chicken/Dongguan/2690/2013_|_A_/_H5N6
A/chicken/Shenzhen/2269/2013_|_A_/_H5N6
A/chicken/Guangxi/04.10_NM140/2015__|_A_/_H5N6
A/duck/Hunan/12.17_YYGK0057/2014__|_A_/_H5N6
A/environment/Zhenjiang/C13/2013_|_A_/_H5N6
A/duck/Vietnam/LBM360c1-4-1/2013_|_A_/_H5N6
A/duck/Guangdong/04.15_SZBAXQ019/2015(Mixed)_|_A_/_H5N6
A/chicken/Guangdong/S1311/2010_|_A_/_H6N6
A/chicken/Jiangxi/04.01__NCDZT0048-O/2015(Mixed)_|_A_/_H5N6
A/Mandarin_duck/Korea/K18-3/2018_|_A_/_H5N6
A/Eurasian_wigeon/Netherlands/1/2018_|_A_/_H5N6
A/jungle_crow/Hyogo/2803A002/2018_|_A_/_H5N6
A/great_crested_grebe/Uvs-Nuur_Lake/341/2016_|_A_/_H5N8
A/Bar-headed_Goose/Qinghai/BTY1-B/2016_|_A_/_H5N8
A/mallard_duck/Netherlands/30/2014_|_A_/_H4N6
A/duck/Mongolia/127/2015_|_A_/_H4N6
A/duck/Eastern_China/S1210/2013_|_A_/_H5N8
A/mallard_duck/Georgia/3/2016_|_A_/_H4N6
A/breeder_duck/Korea/Gochang1/2014_|_A_/_H5N8
A/duck/Jiangsu/k1203/2010_|_A_/_H5N8
A/duck/Hubei/ZYSYG5/2015_|_A_/_H5N6
A/duck/Hubei/ZYSYG18/2015_|_A_/_H5N6
A/common_teal/Korea/KU-12/2015_|_A_/_H5N8
A/duck/Eastern_China/L1021/2012_|_A_/_H5N8
A/duck/Hunan/S2046/2011_|_A_/_H4N2
7.0
1940
1960
1980
2000
2020

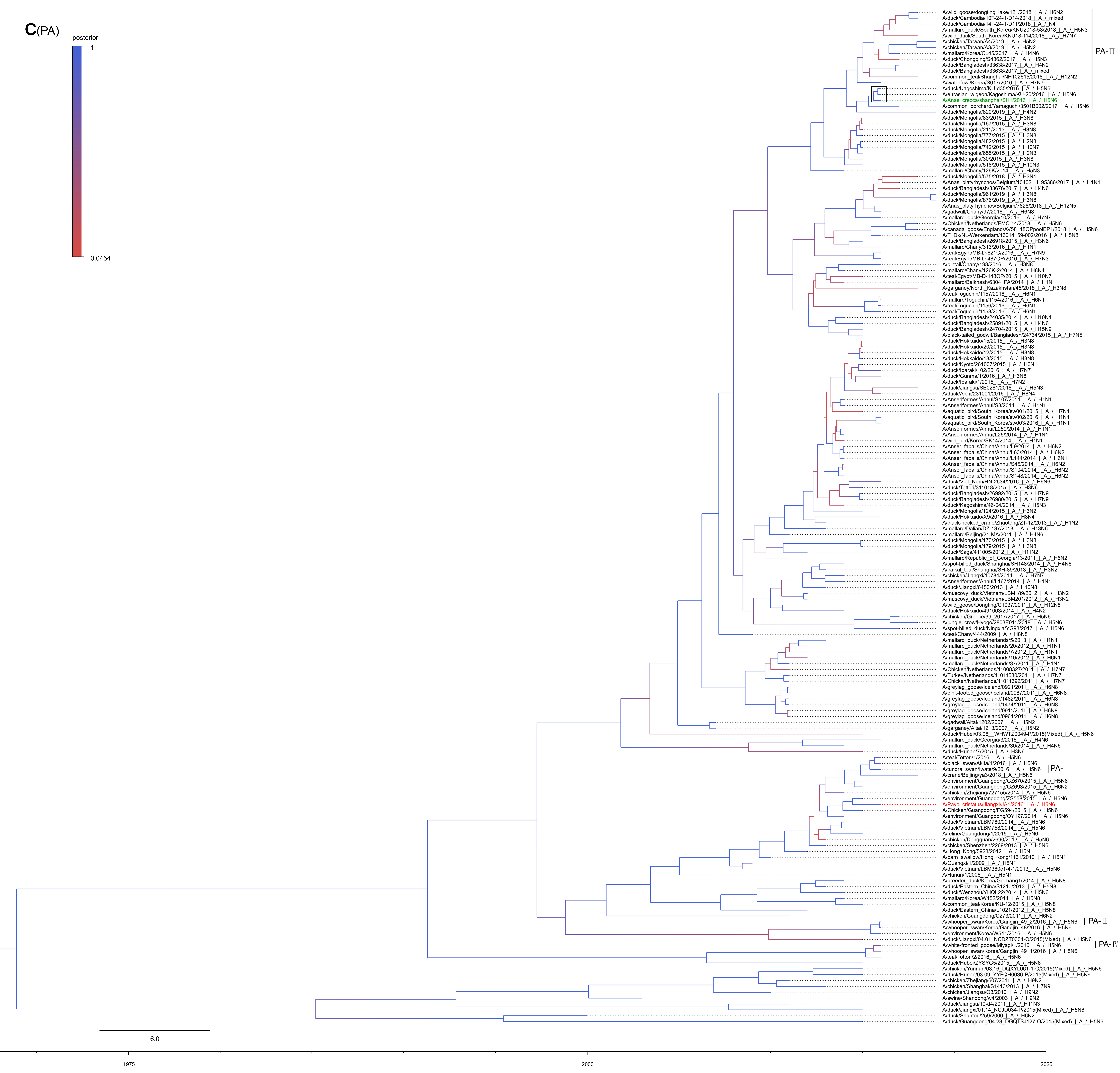
C(PA)
posterior
1
0.0454
PA-Ⅲ
PA-Ⅰ
PA-Ⅱ
PA-Ⅳ
6.0
1975
2000
2025

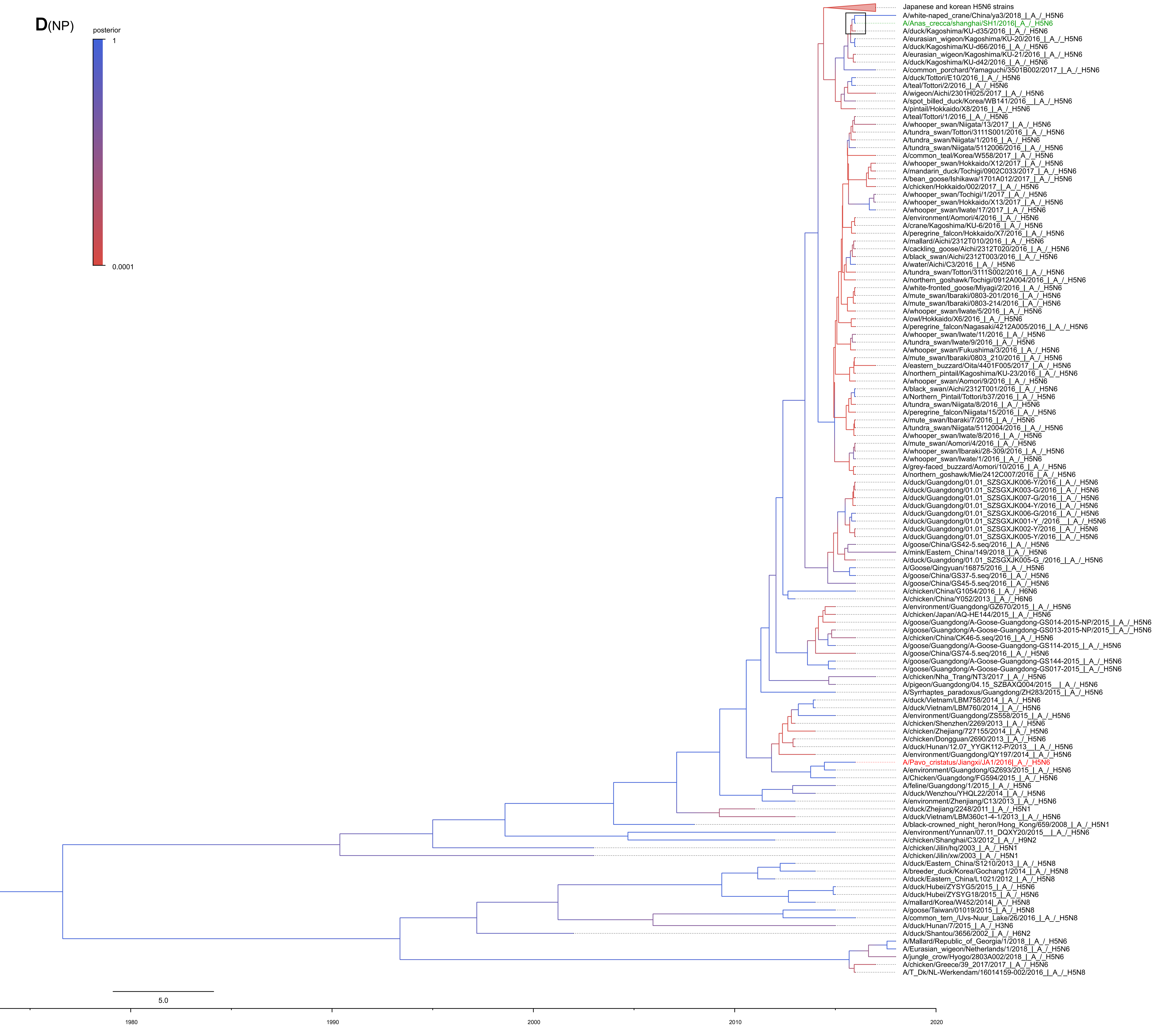
D(NP)
posterior
1
0.0001
Japanese and korean H5N6 strains
A/white-naped_crane/China/ya3/2018_|_A_/_H5N6
A/Anas_crecca/shanghai/SH1/2016|_A_/_H5N6
A/duck/Kagoshima/KU-d35/2016_|_A_/_H5N6
A/eurasian_wigeon/Kagoshima/KU-20/2016_|_A_/_H5N6
A/duck/Kagoshima/KU-d66/2016_|_A_/_H5N6
A/eurasian_wigeon/Kagoshima/KU-21/2016_|_A_/_H5N6
A/duck/Kagoshima/KU-d42/2016_|_A_/_H5N6
A/common_porchard/Yamaguchi/3501B002/2017_|_A_/_H5N6
A/duck/Tottori/E10/2016_|_A_/_H5N6
A/teal/Tottori/2/2016_|_A_/_H5N6
A/wigeon/Aichi/2301H025/2017_|_A_/_H5N6
A/spot_billed_duck/Korea/WB141/2016__|_A_/_H5N6
A/pintail/Hokkaido/X8/2016_|_A_/_H5N6
A/teal/Tottori/1/2016_|_A_/_H5N6
A/whooper_swan/Niigata/13/2017_|_A_/_H5N6
A/tundra_swan/Tottori/3111S001/2016_|_A_/_H5N6
A/tundra_swan/Niigata/1/2016_|_A_/_H5N6
A/tundra_swan/Niigata/5112006/2016_|_A_/_H5N6
A/common_teal/Korea/W558/2017_|_A_/_H5N6
A/whooper_swan/Hokkaido/X12/2017_|_A_/_H5N6
A/mandarin_duck/Tochigi/0902C033/2017_|_A_/_H5N6
A/bean_goose/Ishikawa/1701A012/2017_|_A_/_H5N6
A/chicken/Hokkaido/002/2017_|_A_/_H5N6
A/whooper_swan/Tochigi/1/2017_|_A_/_H5N6
A/whooper_swan/Hokkaido/X13/2017_|_A_/_H5N6
A/whooper_swan/Iwate/17/2017_|_A_/_H5N6
A/environment/Aomori/4/2016_|_A_/_H5N6
A/crane/Kagoshima/KU-6/2016_|_A_/_H5N6
A/peregrine_falcon/Hokkaido/X7/2016_|_A_/_H5N6
A/mallard/Aichi/2312T010/2016_|_A_/_H5N6
A/cackling_goose/Aichi/2312T020/2016_|_A_/_H5N6
A/black_swan/Aichi/2312T003/2016_|_A_/_H5N6
A/water/Aichi/C3/2016_|_A_/_H5N6
A/tundra_swan/Tottori/3111S002/2016_|_A_/_H5N6
A/northern_goshawk/Tochigi/0912A004/2016_|_A_/_H5N6
A/white-fronted_goose/Miyagi/2/2016_|_A_/_H5N6
A/mute_swan/Ibaraki/0803-201/2016_|_A_/_H5N6
A/mute_swan/Ibaraki/0803-214/2016_|_A_/_H5N6
A/whooper_swan/Iwate/5/2016_|_A_/_H5N6
A/owl/Hokkaido/X6/2016_|_A_/_H5N6
A/peregrine_falcon/Nagasaki/4212A005/2016_|_A_/_H5N6
A/whooper_swan/Iwate/11/2016_|_A_/_H5N6
A/tundra_swan/Iwate/9/2016_|_A_/_H5N6
A/whooper_swan/Fukushima/3/2016_|_A_/_H5N6
A/mute_swan/Ibaraki/0803_210/2016_|_A_/_H5N6
A/eastern_buzzard/Oita/4401F005/2017_|_A_/_H5N6
A/northern_pintail/Kagoshima/KU-23/2016_|_A_/_H5N6
A/whooper_swan/Aomori/9/2016_|_A_/_H5N6
A/black_swan/Aichi/2312T001/2016_|_A_/_H5N6
A/Northern_Pintail/Tottori/b37/2016_|_A_/_H5N6
A/tundra_swan/Niigata/8/2016_|_A_/_H5N6
A/peregrine_falcon/Niigata/15/2016_|_A_/_H5N6
A/mute_swan/Ibaraki/7/2016_|_A_/_H5N6
A/tundra_swan/Niigata/5112004/2016_|_A_/_H5N6
A/whooper_swan/Iwate/8/2016_|_A_/_H5N6
A/mute_swan/Aomori/4/2016_|_A_/_H5N6
A/whooper_swan/Ibaraki/28-309/2016_|_A_/_H5N6
A/whooper_swan/Iwate/1/2016_|_A_/_H5N6
A/grey-faced_buzzard/Aomori/10/2016_|_A_/_H5N6
A/northern_goshawk/Mie/2412C007/2016_|_A_/_H5N6
A/duck/Guangdong/01.01_SZSGXJK006-Y/2016_|_A_/_H5N6
A/duck/Guangdong/01.01_SZSGXJK003-G/2016_|_A_/_H5N6
A/duck/Guangdong/01.01_SZSGXJK007-G/2016_|_A_/_H5N6
A/duck/Guangdong/01.01_SZSGXJK004-Y/2016_|_A_/_H5N6
A/duck/Guangdong/01.01_SZSGXJK006-G/2016_|_A_/_H5N6
A/duck/Guangdong/01.01_SZSGXJK001-Y_/2016__|_A_/_H5N6
A/duck/Guangdong/01.01_SZSGXJK002-Y/2016_|_A_/_H5N6
A/duck/Guangdong/01.01_SZSGXJK005-Y/2016_|_A_/_H5N6
A/goose/China/GS42-5.seq/2016_|_A_/_H5N6
A/mink/Eastern_China/149/2018_|_A_/_H5N6
A/duck/Guangdong/01.01_SZSGXJK005-G_/2016_|_A_/_H5N6
A/Goose/Qingyuan/16875/2016_|_A_/_H5N6
A/goose/China/GS37-5.seq/2016_|_A_/_H5N6
A/goose/China/GS45-5.seq/2016_|_A_/_H5N6
A/chicken/China/G1054/2016_|_A_/_H6N6
A/chicken/China/Y052/2013_|_A_/_H6N6
A/environment/Guangdong/GZ670/2015_|_A_/_H5N6
A/chicken/Japan/AQ-HE144/2015_|_A_/_H5N6
A/goose/Guangdong/A-Goose-Guangdong-GS014-2015-NP/2015_|_A_/_H5N6
A/goose/Guangdong/A-Goose-Guangdong-GS013-2015-NP/2015_|_A_/_H5N6
A/chicken/China/CK46-5.seq/2016_|_A_/_H5N6
A/goose/Guangdong/A-Goose-Guangdong-GS114-2015_|_A_/_H5N6
A/goose/China/GS74-5.seq/2016_|_A_/_H5N6
A/goose/Guangdong/A-Goose-Guangdong-GS144-2015_|_A_/_H5N6
A/goose/Guangdong/A-Goose-Guangdong-GS017-2015_|_A_/_H5N6
A/chicken/Nha_Trang/NT3/2017_|_A_/_H5N6
A/pigeon/Guangdong/04.15_SZBAXQ004/2015__|_A_/_H5N6
A/Syrrhaptes_paradoxus/Guangdong/ZH283/2015_|_A_/_H5N6
A/duck/Vietnam/LBM758/2014_|_A_/_H5N6
A/duck/Vietnam/LBM760/2014_|_A_/_H5N6
A/environment/Guangdong/ZS558/2015_|_A_/_H5N6
A/chicken/Shenzhen/2269/2013_|_A_/_H5N6
A/chicken/Zhejiang/727155/2014_|_A_/_H5N6
A/chicken/Dongguan/2690/2013_|_A_/_H5N6
A/duck/Hunan/12.07_YYGK112-P/2013__|_A_/_H5N6
A/environment/Guangdong/QY197/2014_|_A_/_H5N6
A/Pavo_cristatus/Jiangxi/JA1/2016|_A_/_H5N6
A/environment/Guangdong/GZ693/2015_|_A_/_H5N6
A/Chicken/Guangdong/FG594/2015_|_A_/_H5N6
A/feline/Guangdong/1/2015_|_A_/_H5N6
A/duck/Wenzhou/YHQL22/2014_|_A_/_H5N6
A/environment/Zhenjiang/C13/2013_|_A_/_H5N6
A/duck/Zhejiang/2248/2011_|_A_/_H5N1
A/duck/Vietnam/LBM360c1-4-1/2013_|_A_/_H5N6
A/black-crowned_night_heron/Hong_Kong/659/2008_|_A_/_H5N1
A/environment/Yunnan/07.11_DQXY20/2015__|_A_/_H5N6
A/chicken/Shanghai/C3/2012_|_A_/_H9N2
A/chicken/Jilin/hq/2003_|_A_/_H5N1
A/chicken/Jilin/xw/2003_|_A_/_H5N1
A/duck/Eastern_China/S1210/2013_|_A_/_H5N8
A/breeder_duck/Korea/Gochang1/2014_|_A_/_H5N8
A/duck/Eastern_China/L1021/2012_|_A_/_H5N8
A/duck/Hubei/ZYSYG5/2015_|_A_/_H5N6
A/duck/Hubei/ZYSYG18/2015_|_A_/_H5N6
A/mallard/Korea/W452/2014|_A_/_H5N8
A/goose/Taiwan/01019/2015_|_A_/_H5N8
A/common_tern_/Uvs-Nuur_Lake/26/2016_|_A_/_H5N8
A/duck/Hunan/7/2015_|_A_/_H3N6
A/duck/Shantou/3656/2002_|_A_/_H6N2
A/Mallard/Republic_of_Georgia/1/2018_|_A_/_H5N6
A/Eurasian_wigeon/Netherlands/1/2018_|_A_/_H5N6
A/jungle_crow/Hyogo/2803A002/2018_|_A_/_H5N6
A/chicken/Greece/39_2017/2017_|_A_/_H5N6
A/T_Dk/NL-Werkendam/16014159-002/2016_|_A_/_H5N8
5.0
1980
1990
2000
2010
2020

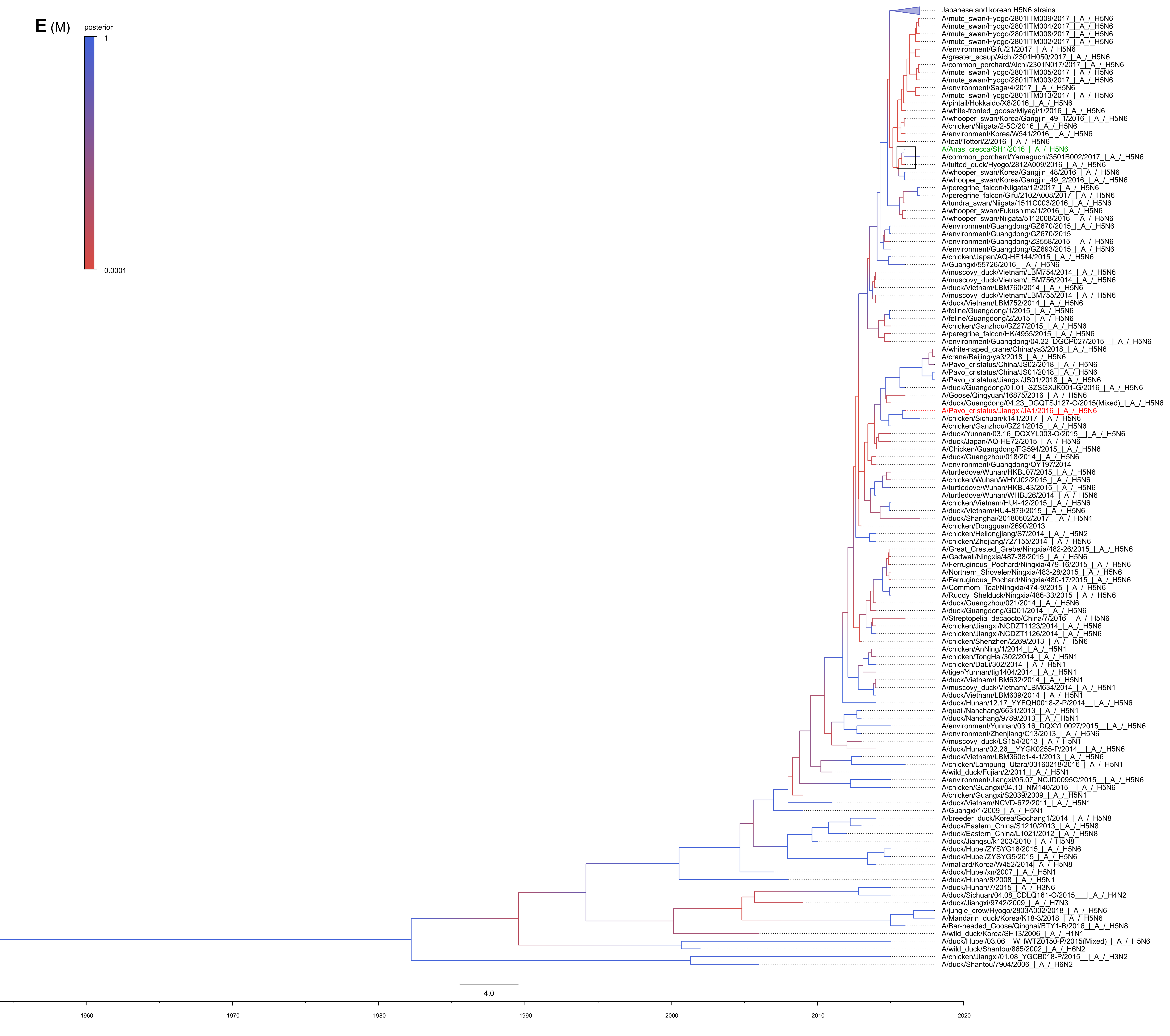

E (M)
posterior
1
0.0001
Japanese and korean H5N6 strains
A/mute_swan/Hyogo/2801ITM009/2017_|_A_/_H5N6
A/mute_swan/Hyogo/2801ITM004/2017_|_A_/_H5N6
A/mute_swan/Hyogo/2801ITM008/2017_|_A_/_H5N6
A/mute_swan/Hyogo/2801ITM002/2017_|_A_/_H5N6
A/environment/Gifu/21/2017_|_A_/_H5N6
A/greater_scaup/Aichi/2301H050/2017_|_A_/_H5N6
A/common_porchard/Aichi/2301N017/2017_|_A_/_H5N6
A/mute_swan/Hyogo/2801ITM005/2017_|_A_/_H5N6
A/mute_swan/Hyogo/2801ITM003/2017_|_A_/_H5N6
A/environment/Saga/4/2017_|_A_/_H5N6
A/mute_swan/Hyogo/2801ITM013/2017_|_A_/_H5N6
A/pintail/Hokkaido/X8/2016_|_A_/_H5N6
A/white-fronted_goose/Miyagi/1/2016_|_A_/_H5N6
A/whooper_swan/Korea/Gangjin_49_1/2016_|_A_/_H5N6
A/chicken/Niigata/2-5C/2016_|_A_/_H5N6
A/environment/Korea/W541/2016_|_A_/_H5N6
A/teal/Tottori/2/2016_|_A_/_H5N6
A/Anas_crecca/SH1/2016_|_A_/_H5N6
A/common_porchard/Yamaguchi/3501B002/2017_|_A_/_H5N6
A/tufted_duck/Hyogo/2812A009/2016_|_A_/_H5N6
A/whooper_swan/Korea/Gangjin_48/2016_|_A_/_H5N6
A/whooper_swan/Korea/Gangjin_49_2/2016_|_A_/_H5N6
A/peregrine_falcon/Niigata/12/2017_|_A_/_H5N6
A/peregrine_falcon/Gifu/2102A008/2017_|_A_/_H5N6
A/tundra_swan/Niigata/1511C003/2016_|_A_/_H5N6
A/whooper_swan/Fukushima/1/2016_|_A_/_H5N6
A/whooper_swan/Niigata/5112008/2016_|_A_/_H5N6
A/environment/Guangdong/GZ670/2015_|_A_/_H5N6
A/environment/Guangdong/GZ670/2015
A/environment/Guangdong/ZS558/2015_|_A_/_H5N6
A/environment/Guangdong/GZ693/2015_|_A_/_H5N6
A/chicken/Japan/AQ-HE144/2015_|_A_/_H5N6
A/Guangxi/55726/2016_|_A_/_H5N6
A/muscovy_duck/Vietnam/LBM754/2014_|_A_/_H5N6
A/muscovy_duck/Vietnam/LBM756/2014_|_A_/_H5N6
A/duck/Vietnam/LBM760/2014_|_A_/_H5N6
A/muscovy_duck/Vietnam/LBM755/2014_|_A_/_H5N6
A/duck/Vietnam/LBM752/2014_|_A_/_H5N6
A/feline/Guangdong/1/2015_|_A_/_H5N6
A/feline/Guangdong/2/2015_|_A_/_H5N6
A/chicken/Ganzhou/GZ27/2015_|_A_/_H5N6
A/peregrine_falcon/HK/4955/2015_|_A_/_H5N6
A/environment/Guangdong/04.22_DGCP027/2015__|_A_/_H5N6
A/white-naped_crane/China/ya3/2018_|_A_/_H5N6
A/crane/Beijing/ya3/2018_|_A_/_H5N6
A/Pavo_cristatus/China/JS02/2018_|_A_/_H5N6
A/Pavo_cristatus/China/JS01/2018_|_A_/_H5N6
A/Pavo_cristatus/Jiangxi/JS01/2018_|_A_/_H5N6
A/duck/Guangdong/01.01_SZSGXJK001-G/2016_|_A_/_H5N6
A/Goose/Qingyuan/16875/2016_|_A_/_H5N6
A/duck/Guangdong/04.23_DGQTSJ127-O/2015(Mixed)_|_A_/_H5N6
A/Pavo_cristatus/Jiangxi/JA1/2016_|_A_/_H5N6
A/chicken/Sichuan/k141/2017_|_A_/_H5N6
A/chicken/Ganzhou/GZ21/2015_|_A_/_H5N6
A/duck/Yunnan/03.16_DQXYL003-O/2015__|_A_/_H5N6
A/duck/Japan/AQ-HE72/2015_|_A_/_H5N6
A/Chicken/Guangdong/FG594/2015_|_A_/_H5N6
A/duck/Guangzhou/018/2014_|_A_/_H5N6
A/environment/Guangdong/QY197/2014
A/turtledove/Wuhan/HKBJ07/2015_|_A_/_H5N6
A/chicken/Wuhan/WHYJ02/2015_|_A_/_H5N6
A/turtledove/Wuhan/HKBJ43/2015_|_A_/_H5N6
A/turtledove/Wuhan/WHBJ26/2014_|_A_/_H5N6
A/chicken/Vietnam/HU4-42/2015_|_A_/_H5N6
A/duck/Vietnam/HU4-879/2015_|_A_/_H5N6
A/duck/Shanghai/20180602/2017_|_A_/_H5N1
A/chicken/Dongguan/2690/2013
A/chicken/Heilongjiang/S7/2014_|_A_/_H5N2
A/chicken/Zhejiang/727155/2014_|_A_/_H5N6
A/Great_Crested_Grebe/Ningxia/482-26/2015_|_A_/_H5N6
A/Gadwall/Ningxia/487-38/2015_|_A_/_H5N6
A/Ferruginous_Pochard/Ningxia/479-16/2015_|_A_/_H5N6
A/Northern_Shoveler/Ningxia/483-28/2015_|_A_/_H5N6
A/Ferruginous_Pochard/Ningxia/480-17/2015_|_A_/_H5N6
A/Common_Teal/Ningxia/474-9/2015_|_A_/_H5N6
A/Ruddy_Shelduck/Ningxia/486-33/2015_|_A_/_H5N6
A/duck/Guangzhou/021/2014_|_A_/_H5N6
A/duck/Guangdong/GD01/2014_|_A_/_H5N6
A/Streptopelia_decaocto/China/7/2016_|_A_/_H5N6
A/chicken/Jiangxi/NCDZT1123/2014_|_A_/_H5N6
A/chicken/Jiangxi/NCDZT1126/2014_|_A_/_H5N6
A/chicken/Shenzhen/2269/2013_|_A_/_H5N6
A/chicken/AnNing/1/2014_|_A_/_H5N1
A/chicken/TongHai/302/2014_|_A_/_H5N1
A/chicken/DaLi/302/2014_|_A_/_H5N1
A/tiger/Yunnan/tig1404/2014_|_A_/_H5N1
A/duck/Vietnam/LBM632/2014_|_A_/_H5N1
A/muscovy_duck/Vietnam/LBM634/2014_|_A_/_H5N1
A/duck/Vietnam/LBM639/2014_|_A_/_H5N1
A/duck/Hunan/12.17_YYFQH0018-Z-P/2014__|_A_/_H5N6
A/quail/Nanchang/6631/2013_|_A_/_H5N1
A/duck/Nanchang/9789/2013_|_A_/_H5N1
A/environment/Yunnan/03.16_DQXYL0027/2015__|_A_/_H5N6
A/environment/Zhenjiang/C13/2013_|_A_/_H5N6
A/muscovy_duck/LS154/2013_|_A_/_H5N1
A/duck/Hunan/02.26__YYGK0255-P/2014__|_A_/_H5N6
A/duck/Vietnam/LBM360c1-4-1/2013_|_A_/_H5N6
A/chicken/Lampung_Utara/03160218/2016_|_A_/_H5N1
A/wild_duck/Fujian/2/2011_|_A_/_H5N1
A/environment/Jiangxi/05.07_NCJD0095C/2015__|_A_/_H5N6
A/chicken/Guangxi/04.10_NM140/2015__|_A_/_H5N6
A/chicken/Guangxi/S2039/2009_|_A_/_H5N1
A/duck/Vietnam/NCVD-672/2011_|_A_/_H5N1
A/Guangxi/1/2009_|_A_/_H5N1
A/breeder_duck/Korea/Gochang1/2014_|_A_/_H5N8
A/duck/Eastern_China/S1210/2013_|_A_/_H5N8
A/duck/Eastern_China/L1021/2012_|_A_/_H5N8
A/duck/Jiangsu/k1203/2010_|_A_/_H5N8
A/duck/Hubei/ZYSYG18/2015_|_A_/_H5N6
A/duck/Hubei/ZYSYG5/2015_|_A_/_H5N6
A/mallard/Korea/W452/2014|_A_/_H5N8
A/duck/Hubei/xn/2007_|_A_/_H5N1
A/duck/Hunan/8/2008_|_A_/_H5N1
A/duck/Hunan/7/2015_|_A_/_H3N6
A/duck/Sichuan/04.08_CDLQ161-O/2015___|_A_/_H4N2
A/duck/Jiangxi/9742/2009_|_A_/_H7N3
A/jungle_crow/Hyogo/2803A002/2018_|_A_/_H5N6
A/Mandarin_duck/Korea/K18-3/2018_|_A_/_H5N6
A/Bar-headed_Goose/Qinghai/BTY1-B/2016_|_A_/_H5N8
A/wild_duck/Korea/SH13/2006_|_A_/_H1N1
A/duck/Hubei/03.06__WHWTZ0150-P/2015(Mixed)_|_A_/_H5N6
A/wild_duck/Shantou/865/2002_|_A_/_H6N2
A/chicken/Jiangxi/01.08_YGCB018-P/2015__|_A_/_H3N2
A/duck/Shantou/7904/2006_|_A_/_H6N2
4.0
1960
1970
1980
1990
2000
2010
2020

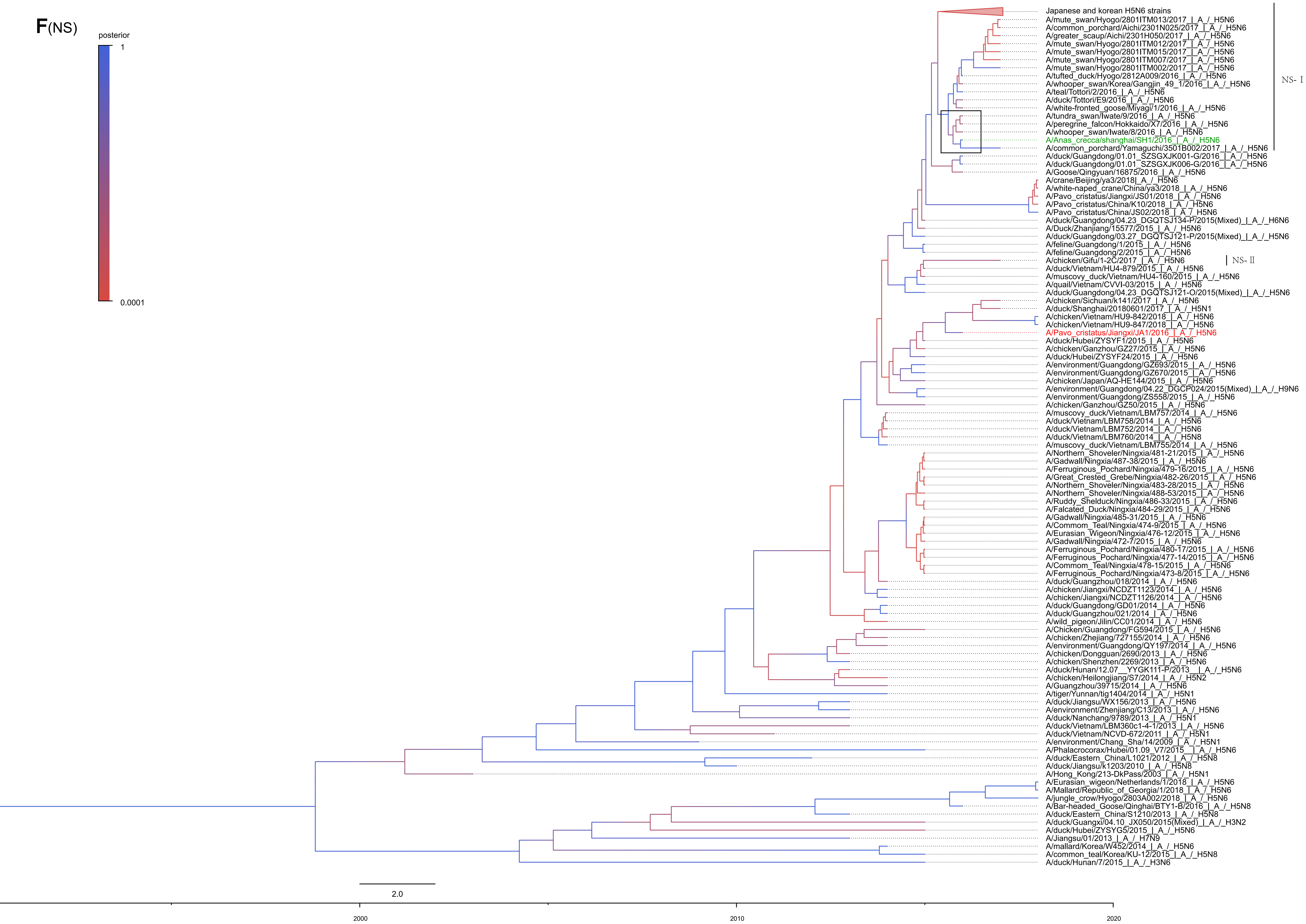
F(NS)
posterior
1
0.0001
Japanese and korean H5N6 strains
A/mute_swan/Hyogo/2801ITM013/2017_|_A_/_H5N6
A/common_porchard/Aichi/2301N025/2017_|_A_/_H5N6
A/greater_scaup/Aichi/2301H050/2017_|_A_/_H5N6
A/mute_swan/Hyogo/2801ITM012/2017_|_A_/_H5N6
A/mute_swan/Hyogo/2801ITM015/2017_|_A_/_H5N6
A/mute_swan/Hyogo/2801ITM007/2017_|_A_/_H5N6
A/mute_swan/Hyogo/2801ITM002/2017_|_A_/_H5N6
A/tufted_duck/Hyogo/2812A009/2016_|_A_/_H5N6
A/whooper_swan/Korea/Gangjin_49_1/2016_|_A_/_H5N6
A/teal/Tottori/2/2016_|_A_/_H5N6
A/duck/Tottori/E9/2016_|_A_/_H5N6
A/white-fronted_goose/Miyagi/1/2016_|_A_/_H5N6
A/tundra_swan/Iwate/9/2016_|_A_/_H5N6
A/peregrine_falcon/Hokkaido/X7/2016_|_A_/_H5N6
A/whooper_swan/Iwate/8/2016_|_A_/_H5N6
A/Anas_crecca/shanghai/SH1/2016_|_A_/_H5N6
A/common_porchard/Yamaguchi/3501B002/2017_|_A_/_H5N6
NS- Ⅰ
A/duck/Guangdong/01.01_SZSGXJK001-G/2016_|_A_/_H5N6
A/duck/Guangdong/01.01_SZSGXJK006-G/2016_|_A_/_H5N6
A/Goose/Qingyuan/16875/2016_|_A_/_H5N6
A/crane/Beijing/ya3/2018|_A_/_H5N6
A/white-naped_crane/China/ya3/2018_|_A_/_H5N6
A/Pavo_cristatus/Jiangxi/JS01/2018_|_A_/_H5N6
A/Pavo_cristatus/China/K10/2018_|_A_/_H5N6
A/Pavo_cristatus/China/JS02/2018_|_A_/_H5N6
A/duck/Guangdong/04.23_DGQTSJ134-P/2015(Mixed)_|_A_/_H6N6
A/Duck/Zhanjiang/15577/2015_|_A_/_H5N6
A/duck/Guangdong/03.27_DGQTSJ121-P/2015(Mixed)_|_A_/_H5N6
A/feline/Guangdong/1/2015_|_A_/_H5N6
A/feline/Guangdong/2/2015_|_A_/_H5N6
A/chicken/Gifu/1-2C/2017_|_A_/_H5N6
NS- Ⅱ
A/duck/Vietnam/HU4-879/2015_|_A_/_H5N6
A/muscovy_duck/Vietnam/HU4-160/2015_|_A_/_H5N6
A/quail/Vietnam/CVVI-03/2015_|_A_/_H5N6
A/duck/Guangdong/04.23_DGQTSJ121-O/2015(Mixed)_|_A_/_H5N6
A/chicken/Sichuan/k141/2017_|_A_/_H5N6
A/duck/Shanghai/20180601/2017_|_A_/_H5N1
A/chicken/Vietnam/HU9-842/2018_|_A_/_H5N6
A/chicken/Vietnam/HU9-847/2018_|_A_/_H5N6
A/Pavo_cristatus/Jiangxi/JA1/2016_|_A_/_H5N6
A/duck/Hubei/ZYSYF1/2015_|_A_/_H5N6
A/chicken/Ganzhou/GZ27/2015_|_A_/_H5N6
A/duck/Hubei/ZYSYF24/2015_|_A_/_H5N6
A/environment/Guangdong/GZ693/2015_|_A_/_H5N6
A/environment/Guangdong/GZ670/2015_|_A_/_H5N6
A/chicken/Japan/AQ-HE144/2015_|_A_/_H5N6
A/environment/Guangdong/04.22_DGCP024/2015(Mixed)_|_A_/_H9N6
A/environment/Guangdong/ZS558/2015_|_A_/_H5N6
A/chicken/Ganzhou/GZ50/2015_|_A_/_H5N6
A/muscovy_duck/Vietnam/LBM757/2014_|_A_/_H5N6
A/duck/Vietnam/LBM758/2014_|_A_/_H5N6
A/duck/Vietnam/LBM752/2014_|_A_/_H5N6
A/duck/Vietnam/LBM760/2014_|_A_/_H5N8
A/muscovy_duck/Vietnam/LBM755/2014_|_A_/_H5N6
A/Northern_Shoveler/Ningxia/481-21/2015_|_A_/_H5N6
A/Gadwall/Ningxia/487-38/2015_|_A_/_H5N6
A/Ferruginous_Pochard/Ningxia/479-16/2015_|_A_/_H5N6
A/Great_Crested_Grebe/Ningxia/482-26/2015_|_A_/_H5N6
A/Northern_Shoveler/Ningxia/483-28/2015_|_A_/_H5N6
A/Northern_Shoveler/Ningxia/488-53/2015_|_A_/_H5N6
A/Ruddy_Shelduck/Ningxia/486-33/2015_|_A_/_H5N6
A/Falcated_Duck/Ningxia/484-29/2015_|_A_/_H5N6
A/Gadwall/Ningxia/485-31/2015_|_A_/_H5N6
A/Commom_Teal/Ningxia/474-9/2015_|_A_/_H5N6
A/Eurasian_Wigeon/Ningxia/476-12/2015_|_A_/_H5N6
A/Gadwall/Ningxia/472-7/2015_|_A_/_H5N6
A/Ferruginous_Pochard/Ningxia/480-17/2015_|_A_/_H5N6
A/Ferruginous_Pochard/Ningxia/477-14/2015_|_A_/_H5N6
A/Commom_Teal/Ningxia/478-15/2015_|_A_/_H5N6
A/Ferruginous_Pochard/Ningxia/473-8/2015_|_A_/_H5N6
A/duck/Guangzhou/018/2014_|_A_/_H5N6
A/chicken/Jiangxi/NCDZT1123/2014_|_A_/_H5N6
A/chicken/Jiangxi/NCDZT1126/2014_|_A_/_H5N6
A/duck/Guangdong/GD01/2014_|_A_/_H5N6
A/duck/Guangzhou/021/2014_|_A_/_H5N6
A/wild_pigeon/Jilin/CC01/2014_|_A_/_H5N6
A/Chicken/Guangdong/FG594/2015_|_A_/_H5N6
A/chicken/Zhejiang/727155/2014_|_A_/_H5N6
A/environment/Guangdong/QY197/2014_|_A_/_H5N6
A/chicken/Dongguan/2690/2013_|_A_/_H5N6
A/chicken/Shenzhen/2269/2013_|_A_/_H5N6
A/duck/Hunan/12.07__YYGK111-P/2013__|_A_/_H5N6
A/chicken/Heilongjiang/S7/2014_|_A_/_H5N2
A/Guangzhou/39715/2014_|_A_/_H5N6
A/tiger/Yunnan/tig1404/2014_|_A_/_H5N1
A/duck/Jiangsu/WX156/2013_|_A_/_H5N6
A/environment/Zhenjiang/C13/2013_|_A_/_H5N6
A/duck/Nanchang/9789/2013_|_A_/_H5N1
A/duck/Vietnam/LBM360c1-4-1/2013_|_A_/_H5N6
A/duck/Vietnam/NCVD-672/2011_|_A_/_H5N1
A/environment/Chang_Sha/14/2009_|_A_/_H5N1
A/Phalacrocorax/Hubei/01.09_V7/2015__|_A_/_H5N6
A/duck/Eastern_China/L1021/2012_|_A_/_H5N8
A/duck/Jiangsu/k1203/2010_|_A_/_H5N8
A/Hong_Kong/213-DkPass/2003_|_A_/_H5N1
A/Eurasian_wigeon/Netherlands/1/2018_|_A_/_H5N6
A/Mallard/Republic_of_Georgia/1/2018_|_A_/_H5N6
A/jungle_crow/Hyogo/2803A002/2018_|_A_/_H5N6
A/Bar-headed_Goose/Qinghai/BTY1-B/2016_|_A_/_H5N8
A/duck/Eastern_China/S1210/2013_|_A_/_H5N8
A/duck/Guangxi/04.10_JX050/2015(Mixed)_|_A_/_H3N2
A/duck/Hubei/ZYSYG5/2015_|_A_/_H5N6
A/Jiangsu/01/2013_|_A_/_H7N9
A/mallard/Korea/W452/2014_|_A_/_H5N6
A/common_teal/Korea/KU-12/2015_|_A_/_H5N8
A/duck/Hunan/7/2015_|_A_/_H3N6
2.0
2000
2010
2020

Supplementary Figure 1. Genesis analysis of SH1 and JA1. The phylogenetic trees were conducted using gene sequences listed in the NCBI. SH1 is marked in green and JA1 is marked in red. Panels (A) to (E) represent PB2, PB1, PA, NP, M, and NS separately. All the trees were built by BEAST (v 1.8.4) and displayed using FigTree (v 1.4.2).

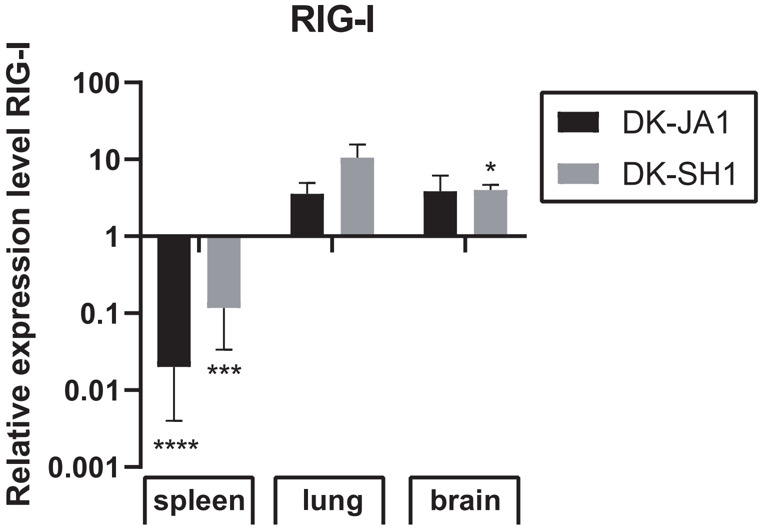

Supplementary Figure 2. Expression profiles of RIG-I in the target tissues of chickens and ducks infected with H5N6. Each bar represents the level of target gene mRNA relative to mock after normalizing to GAPDH. Data is expressed as the mean values ± standard deviation. Statistical analysis was performed with Student's t-test (*p < 0.05, **p < 0.01).

Supplementary Table S1. Dataset size and best-fit model for each gene segment.

Gene segment datasets	Sequence Numbers	Substitution Models	Relax-clock models[a]	Tree Models	MCMC chain length[b]
HA	97	GTR+I	uced	GMRF	100
NA	105	GTR+G	uced	GMRF	100
PB2	164	GTR+I+G	uced	EG	200
PB1	149	GTR+G	uced	BSG	200
PA	173	GTR+G	uced	BSG	100
NP	166	GTR+G	uced	EG	200
M	155	HKY+I+G	uced	EG	100
NS	153	HKY+G	uced	BSG	100

[a] uced: uncorrelated exponential clock model

[b] The unit was million.

Supplementary Table S2. Groups of chickens (number).

	virus	JA1		SH1		
	Observation days	2[a]	14	2[a]	14	Total number
Groups	Control (PBS)	3	3	3	3	12
	treatment	3	5	3	5	16
	Contact (naive)		2		2	4
	Total number					32

[a]At 2 DPI, three chickens from treatment group and three chickens from control groups were humanely euthanized.

Supplementary Table S3. Groups of ducks (number).

	virus	JA1		SH1		
	Observation days	2[a]	14	2[a]	14	Total number
Groups	Control (PBS)	3	3	3	3	12
	treatment	3	5	3	5	16
	Contact (naive)		2		2	4
	Total number					32

[a]At 2 DPI, three ducks from treatment group and three ducks from control groups were humanely euthanized.

Supplementary Table S4. Primer sequences used in the study**.**

Primer name	Sequence (5'→3')	Product size (bp)	accession no.
C-GAPDH	F: CCTCTCTGGCAAAGTCCAAG	200	V00407
C-GAPDH	R: CATCTGCCCATTTGATGTTG		
C-IL-6	F: ATGTGCAAGAAGTTCACCGTG	171	EU170468
C-IL-6	R: TTCCAGGTAGGTCTGAAAGGCGAA		
C-IFN-γ	F: GCTGACGGTGGACCTATTATT	248	DQ906156
C-IFN-γ	R: TGGATTCTCAAGTCGCTCATCG		
C-TLR-7	F: TGTGATGTGGAAGCCTTTGA	218	DQ780342
C-TLR-7	R: ATTATCTTTGGGCCCCAGTC		
D-GAPDH	F: ATGTTCGTGATGGGTGTGAA	176	AY436595
D-GAPDH	R: CTGTCTTCGTGTGTGGCTGT		
D-IL-6	F: TTCGACGAGGAGAAATGCTT	150	AB191038
D-IL-6	R: CCTTATCGTCGTTGCCAGAT		
D-IFNγ	F: GCTGATGGCAATCCTGTTTT	247	AJ012254
D-IFNγ	R: GGATTTTCAAGCCAGTCAGC		
D-TLR-7	F: CCTTTCCCAGAGAGCATTCA	154	AY940195
D-TLR-7	R: TCAAGAAATATCAAGATAATCACATCA		
iNOS	F: CCACCAGGAGATGTTGAATATGTC	75	NM204961/ NM001310393
iNOS	R: CCAGATGTGTGTTTTCCATGCA		

Supplementary Table S5. Gene segments of SH1 demonstrating that it is most closely related to the H5N6 virus that was isolated from Tottori, Japan**.**

Gene	Virus with the highest identity	Identify(%)
HA	A/duck/Tottori/E9/2016_|_A_/_H5N6	99.65%
	A/whooper_swan/Korea/Gangjin_49_1/2016_|_A_/_H5N6	99.65%
NA	A/white-fronted_goose/Miyagi/1/2016_|_A_/_H5N6	99.72%
	A/duck/Tottori/E9/2016_|_A_/_H5N6	99.43%
PB2	A/white-fronted_goose/Miyagi/1/2016_|_A_/_H5N6	99.66%
	A/duck/Tottori/E9/2016_|_A_/_H5N6	99.70%
PB1	A/pintail/Hokkaido/X8/2016_|_A_/_H5N6	99.70%
PA	A/eurasian_wigeon/Kagoshima/KU-20/2016_|_A_/_H5N6	99.87%
	A/duck/Kagoshima/KU-d35/2016_|_A_/_H5N6	99.77%
NP	A/duck/Kagoshima/KU-d35/2016_|_A_/_H5N6	99.87%
M	A/tufted_duck/Hyogo/2812A009/2016_|_A_/_H5N6	99.90%
NS	A/whooper_swan/Iwate/8/2016_|_A_/_H5N6	99.65%
	A/peregrine_falcon/Hokkaido/X7/2016_|_A_/_H5N6	99.77%
	A/tundra_swan/Iwate/9/2016_|_A_/_H5N6	99.77%

Supplementary Table S6 Relative expression levels of immune gene in the spleen of chickens and ducks.

Virus	SH1	SH1		JA1	JA1	
Species	Ducks (n=3)	Chickens (n=3)	P[a]	Ducks (n=3)	Chicken (n=3)	P[a]
Tissue	Spleen	Spleen		Spleen	Spleen	
Mortality	Not Fatal (0%)	Fatal (100%)		Fatal (80%)	Fatal (100%)	
TLR-7	8.34±3.76	6.11±5.62	0.662678	20.88±23.28	31.46±31.98	0.667504
TLR-3	0.00±0.00	6.05±4.11	0.17316	0.08±0.1	3.7±0.9	0.0049
MDA5	0.00±0.00	43.13±0.24	0.000015	0.06±0.09	48.15±32.22	0.066079
TNF-α	2.52±1.58	5.9±1.16	0.067382	13.05±15.57	8.13±6.27	0.718475
IL-1β	0.24±0.11	64.69±53.84	0.20661	59.72±4.06	67.64±28.98	0.739134
IL-6	2.14±1.15	2.36±0.17	0.765517	8.25±0.07	17.03±1.72	0.0064
IFN-γ	0.67±0.31	98.91±20.43	0.001136	41.65±12.76	23.17±12.94	0.153003
i-NOS	0.44±0.01	7.71±3.14	0.052964	51.65±28.8	11.79±11.02	0.088704

Levels are given as mean values of target gene mRNA relative to mock after normalizing to GAPDH Data is expressed as the mean values ± standard deviation. Statistical analysis was performed with Student's t-test [a]Comparison between chickens and ducks.

Supplementary Table S7 Relative expression levels of immune gene in the lung of chickens and ducks.

Virus	SH1	SH1		JA1	JA1	
Species	Ducks (n=3)	Chickens (n=3)	P[a]	Ducks (n=3)	Chicken (n=3)	P[a]
Tissue	Lung	Lung		Lung	Lung	
Mortality	Not Fatal (0%)	Fatal (100%)		Fatal (80%)	Fatal (100%)	
TLR-7	24.63±9.47	3.22±0.18	0.085528	0.38±0.36	45.97±5.45	0.007092
TLR-3	1.41±0.56	8.48±4.16	0.235375	3.84±0.01	0.39±0.34	0.079936
MDA5	1.51±0.61	19.89±4.29	0.010576	5.55±6.06	2.38±2.05	0.435579
TNF-α	0.38±0.19	4.51±0.15	0.001687	0.25±0.33	10.39±11.51	0.332062
IL-1β	2.25±0.47	4.79±1.7	0.143753	19.02±17.19	75.16±3.86	0.045854
IL-6	2.44±1.89	15.84±5.35	0.023829	0.53±0.55	29.89±5.64	0.006074
IFN-γ	3.06±2.33	36.35±1.21	0.000375	0.38±0.36	1.74±0.14	0.038343
i-NOS	16.81±1.8	39.88±4.28	0.001003	0.07±0.08	0.09±0.09	0.817409

Similar to Table S6 for lung. [a]Comparison between chickens and ducks.

Supplementary Table S8 Relative expression levels of immune gene in the brain of chickens and ducks.

Virus	SH1	SH1		JA1	JA1	
Species	Ducks (n=3)	Chickens (n=3)	P[a]	Ducks (n=3)	Chicken (n=3)	P[a]
Tissue	Brain	Brain		Brain	Brain	
Mortality	Not Fatal (0%)	Fatal (100%)		Fatal (80%)	Fatal (100%)	
TLR-7	8.34±3.76	0.88±0.14	0.0174	10.59±0.93	7.36±3.09	0.165622
TLR-3	0.00±0.00	74.61±32.42	0.087554	5.91±0.39	76.83±9.13	0.008194
MDA5	3.85±1.17	440.76±208.89	0.097809	8.83±4.18	296.66±13.76	0.000045
TNF-α	4.77±1.4	0.52±0.34	0.00693	25.27±32.39	0.09±0.15	0.236653
IL-1β	1.41±0.35	37.56±3.59	0.0049	14.42±9.08	31.06±15.34	0.27204
IL-6	43.25±10.6	3.58±1.29	0.115	20.12±13.54	59.87±24.06	0.131566
IFN-γ	1.87±1.22	3.77±2.74	0.34682	8.59±1.44	4.06±2.24	0.09009
i-NOS	5.32±0.39	2.08±0.23	0.128	146.4±3.74	6.82±0.78	0.000007

Similar to Table S9 for brain. [a]Comparison between chickens and ducks.

Supplementary Table S9 Relative expression levels of immune gene in the spleen and lung of ducks.

Virus	SH1	JA1	P[a]	SH1	JA1	P[a]
Species	Ducks (n=3)	Ducks (n=3)		Ducks (n=3)	Ducks (n=3)	
Tissue	Spleen	Spleen		Lung	Lung	
Mortality	Not Fatal (0%)	Fatal (80%)		Not Fatal (0%)	Fatal (80%)	
TLR7	8.34±3.76	20.88±23.28	0.524602	24.63±9.47	0.38±0.36	0.068646
TLR3	0.00±0.00	0.08±0.1	0.370349	1.41±0.56	3.84±0.01	0.025856
RIG-I	0.12±0.14	0.06±0.09	0.435117	10.53±8.86	3.56±1.41	0.249468
MDA5	0.00±0.00	0.02±0.02	0.455521	1.51±0.61	5.55±6.06	0.4472
TNFα	2.52±1.58	13.05±15.57	0.441721	0.38±0.19	0.25±0.33	0.663971
IL-1β	0.24±0.11	59.72±4.06	0.00232	2.25±0.47	19.02±17.19	0.301802
IL-6	2.14±1.15	8.25±0.07	0.005707	2.44±1.89	0.53±0.55	0.275846
IFNγ	0.67±0.31	41.65±12.76	0.00512	3.06±2.33	0.38±0.36	0.223593
iNOS	0.44±0.01	51.65±28.8	0.097107	16.81±1.8	0.07±0.08	0.001112

Levels are given as mean values of target gene mRNA relative to mock after normalizing to GAPDH

Supplementary Table S10 Relative expression levels of immune gene in the brain of ducks.

Virus	SH1	JA1	P[a]
Species	Ducks (n=3)	Ducks (n=3)	
Tissue	Brain	Brain	
Mortality	Not Fatal (0%)	Fatal (80%)	
TLR7	8.34±3.76	10.59±0.93	0.004398
TLR3	0.00±0.00	5.91±0.39	0.041364
RIG-I	3.85±1.17	3.86±2.31	0.939418
MDA5	3.85±1.17	8.83±4.18	0.214794
TNFα	4.77±1.4	25.27±32.39	0.316613
IL-1β	1.41±0.35	14.42±9.08	0.179952
IL-6	43.25±10.6	20.12±13.54	0.197596
IFNγ	1.87±1.22	8.59±1.44	0.010819
iNOS	5.32±0.39	146.4±3.74	0.000006

Similar to Table S9 for brain. [a]Comparison between JA1 and SH1.

Supplementary Table S11 Amino acid differences of whole gene segments between H5N6 HPAIVs, JA1 and SH1.

Protein	Amino Acid Position	JA1	SH1	Function[a]	Reference
PB2	2	D	N	1-35.PB1-interacting-region.N-terminal 35 amino acid region of PB2 forms an interface with the C-terminal three helix bundle of PB1 (aa 685-757).	PubMed: 19461581
PB2	178	T	A		
PB2	221	A	T		
PB2	441	D	N	320-483 PB2_cap-binding-site.This fragment is a domain co-crystalized with m7GTP.	PubMed: 18454157
PB1	12	V	I	1-25 PA-binding-region The N-terminal region of PB1 interacts with the C-terminus of PA (residues	PubMed: 18615018,
PB1	14	V	A		
PB1	54	E	K		
PB1	108	L	I		
PB1	149	I	V		
PB1	157	A	T		
PB1	178	V	G		
PB1	200	V	I		
PB1	298	L	V		
PB1	386	R	K		
PB1	451	I	V		
PB1	517	I	V		
PB1	635	R	K		
PB1	644	I	V		
PB1	654	G	S		
PB1	655	I	M		
PB1	746	I	T	685-757,PB2-binding-region,The C-terminal three helix bundle of PB1 binds to 1–37 and 1–86 fragments on the N-terminus of PB2. This interface is crucial for the regulation of overall enzyme	
PB1-F2	2-39	DEL	complete		
PB1-F2	35	DEL	S	S35.PKC phosphorylation site is related to proapoptotic activity of the PB1-F2 in PR8 strains.	PubMed: 19523156
PB1-F2	46	T	M		
PB1-F2	47	S	N		
PB1-F2	48	P	Q		
PB1-F2	50	G	D		
PB1-F2	52	R	H		
PB1-F2	57	C	Y		
PB1-F2	62	L	P		PubMed: 18005742
PB1-F2	67	P	L		
PB1-F2	71	S	Y		
PB1-F2	74	I	T		
PB1-F2	75	R	H		
PB1-F2	76	A	V		
PB1-F2	83	S	F		
PB1-F2	84	N	S		

PB1-F2	62-70	LSLKNPIQE	PSLKNLIQ	determinants-of-disease-progression.Immunizing mice with a synthetic peptide corresponding to positions 62-70 of PB1-F2 is recognized by CD8+ T-lymphocytes that lyse virus-infected cells.	PubMed: 11726970
	61-87	WLSLKNPIQESLKIRALKRWKLSNKQE	WPSLKNLIQEYLKTHVLKRWKLFSKQE	W61,P62,S63,L64,K65,N66,L67,I68,Q69,E70,Y71,L72,K73,T74,H75,V76,L77,K78,R79,W80,K81,L82,F83,S84,K85,Q86,E87 (SH1).determinant-of-secondary-bacterial-infections. Enhancement of secondary bacterial pneumonia in mice is seen by pretreatment with PB1-F2 C-terminus derived peptides .	PubMed: 18005742
PB1-F2	89	T	I		
PA	20	T	A		
PA	27	S	D		
PA	63	A	V		
PA	101	D	E		
PA	142	R	N		
PA	261	M	L		
PA	259-261	PFM	PFL	Inflammatory-response_259(3)_259-261_CCR5-signaling-induced.Among 1918 WT and 1918 FS there is no apparent change in histopathological changes. Gene ontology analysis indicated that sequences showing differential expression between 1918 WT and 1918 FS infection were associated predominantly with inflammation or immune response, apoptosis, cell differentiation, tissue remodeling. Genes in CCR5 signaling in macrophages is more highly induced in 1918 FS and 1918 PTC mutants relative to WT.	PubMed: 22745253
PA	321	N	I		
PA	327	D	E		
PA	352	D	E		
PA	391	K	R		
PA	404	S	A		
PA	407	V	I		
PA	492	R	K		
PA	538	V	E		
PA	614	T	N		
PA	631	S	G	determinant-of-virulence.Passaging of HK156 viruses in mouse brain and embryonated eggs led to the selection of high and low virulent variants in the mice model respectively. These phenotypic changes are confered by changes in amino acids in HA residues (211), PB1 (456 and 712), NP (127) and NS1 (101) proteins, together with the PA (631)	PubMed: 10873787
PA	683	L	H		
PA	688	D	E		

PA	257-716			PB1-binding-region.The C-terminus of PA interacts with the N-terminal region of PB1 (residues 1-25). This subunit interface complex is essential for initiation of transcription.	PubMed: 18615018
PA-X	20	T	A		
PA-X	27	S	D		
PA-X	63	A	V		
PA-X	101	D	E		
PA-X	142	R	N		
PA-X	193	N	S		
PA-X	204	G	D		
PA-X	215	L	P		
PA-X	228	I	T		
PA-X	248	K	R		
PA-X	251	R	K		
HA	-8	S	A	-1--16.signal-peptide.This region denotes a potential N-terminal signal peptide	
HA	40	K	R		
HA	83	A	T		
HA	126	T	E		
HA	97,108,126,138,212,217	DITQKS	DIEQKS	determinants-of-pathogenicity.Recombinants with changed HA residues: 97, 108, 126, and 138 reduced the pathogenicity of the highly pathogenic A/chicken/Hong Kong/YU562/01 virus and increased the pathogenicity of the mildly pathogenic A/goose/Hong Kong/437-10/99 virus.The substitutions E212K, and P217S resulted in increased pathogenicity of both high and moderately pathogenic viruses.	PubMed: 15331729
HA	127	S	T		
HA	140	M	V		
HA	162	M	K		
HA	169	R	G		
HA	185	E	A		
HA	223	R	Q	species-adaptation_239(1)_239N_Increased-binding-to-alpha2-6.Introduction of Ser223Asn substitution in the A/Vietnam/1203/2004 backbone conferred increased binding to 6' sialyl lactosamine relative to WT parental virus using ELISA based	PubMed:16226289, PubMed:20130132, PubMed:20392847
HA	234	R	K		
HA	259	T	K		
HA	155,156,223	DAR	DAQ	determinant-of-virulence NAN_Decreased-virulence.Introduction of NAN substitutions in the A/Vietnam/1203/2004 backbone conferred increased affinity for alpha2-6SAL using solid phase assay. The mutant virus showed 100 fold reduction in the lethality of WT.	PubMed:19116267

HA	183,223	NR	NQ	GN_Increased-binding-to-alpha2-6.Introduction of GN in the A/Vietnam/1203/2004 backbone conferred increased binding to alpha 2-6 while retaining strong preference for alpha 2-3 sialoglycans using glycan array analysis.	PubMed:20392847
HA	182,192, 222,223, 224	NKQRG	NKQQG	KRLNS_Increased-binding-to-alpha2-6.Introduction of KRLNS substitutions in the A/Indonesia/5/2005 backbone agglutinated alpha 2-6 and retained affinity for alpha 2-3 in shown using hemagglutination assay with modified turkey	PubMed:20392847
HA	273	N	H		
HA	276	A	T		
NP	51	D	G	1-77.RNA-binding-subregion	
NP	167	V	M	79-180.RNA-binding-subregion	
NP	430	A	T	340-498.PB2-binding-site;371-465.NP-association-region;255-465.PB2-interaction-domain	PubMed: 9621005 10405371 9621005
NA	27	A	T		
NA	45	I	M		
NA	50	T	M		
NA	76	V	M		
NA	249	N	S		
NA	262	M	K		
NA	397	I	V		
M1	168	I	T	1-252.RNP-binding-region.This C-terminal region binds to vRNP.	UniProt: P03485 , PubMed: 11222100
M1	227	A	T		
M2	13	S	N		
M2	50	Y	C	The cysteine residue at this site is is responsible for thio-ether linkage of the fatty acid. palmitoylation. The viruses lacking the palmitoylation site at this residue have been shown to cause a modest reduction in virulence in vivo (mouse models) although the effect is not seen tissue culture cells;46-60.CRAC-motif.This is the 'cholesterol recognition consensus (CRAC) motif' found downstream of the transmembrane domain in the cytoplasmic tail region and also possess the C50 palmitoylation site. It is involved in M2 cholesterol binding.	PubMed:7529332, 19553312,15221235
NS1	65	M	V	1-73.RNA-binding-domain.The N-terminal domain of NS1 binds several RNA species, including dsRNA. This domain also mediates interactions with RIG-I, possibly via dsRNA intermediates, PABPI, and importin-alpha.	PubMed: 18796704, 17475623, 18813227
NS1	70	D	N		
NS1	76	A	T	74-86.inter-domain-linker.Flexible linker between the RNA binding and effector domains. Can be variable in length, with a 5aa deletion commonly reported in recent H5N1 isolates.	PubMed: 18987632, UniProt: P03496
NS1	86	A	T	74-86.inter-domain-linker; 81-113.eIF4GI-interaction-site.Interaction of NS1 with eIF4GI may lead to preferential translation of viral mRNAs;	PubMed: 18987632,10938102 UniProt: P03496

NS1	88	R	C	81-113.eIF4GI-interaction-site.Interaction of NS1 with eIF4GI may lead to preferential translation of viral mRNAs;	PubMed: 10938102
NS1	139	G	N	137-147,nuclear-export-signal.Sequence added to a heterologous protein causes nuclear export. L144 and L146 are essential for this activity;87-203,effector-domain.The NS1 effector domain mediates interactions with several host proteins and may stabilize the N-terminal RNA-binding domain.	PubMed: 9560194, UniProt: P03496; PubMed: 18725644, 18796704, 16715094,
NS1	215	P	S	Threonine-215(T-215) is phosphorylated by a subset of proline-directed kinases (e.g. CDKs/ERKs) acting via a defined motif. Required for efficient virus replication in tissue-culture;204-230,flexible-tail.The flexible tail appears to be unstructured and variable in length. It contains a number of motifs, including CDK/ERK phosphorylation, Crk/CrkL SH3 binding, PDZ ligand and NoLS/NLS2; 212,214,215,217,Crk/CrkL-SH3-binding-site.Crk/CrL-SH3 binding motif commonly found in avian influenza A virus strains as well as in the human 1918 pandemic virus. Shown to be required for Crk/CrkL binding.	PubMed: 19007960; PubMed: 18585749, UniProt: P03496; PubMed: 18165234
NS1	230	V	I	204-230,flexible-tail.The flexible tail appears to be unstructured and variable in length. It contains a number of motifs, including CDK/ERK phosphorylation, Crk/CrkL SH3 binding, PDZ ligand and NoLS/NLS2;223-237,PABPII-binding-site.Refers to poly(A)-binding protein II (PABPII)-binding region of NS1. May be involved in inhibiting the posttranscriptional 3'-end processing of cellular pre-mRNAs;226-230,Tissue-tropism.	PubMed: 18585749, UniProt: P03496; PubMed: 10205180, 11421366
NS1	226-230	IESEV	IESEI	Tissue-tropism_226(5)_ESEV/EPEV_Histologic-alteration; Clinical-symptoms-of-disease_226(5)_ESEV/EPEV_Significant-weight-loss.Introduction of the PL motif at the C terminal in the virus A/WSN/33 conferred significant weight loss compared to WT. The virus variant showed severe alveolitis and hemorrhage in lung tissue of mice.	PubMed: 18334632
NS1	227-230	ESEV	ESEI	227-230 PDZ-ligand-motif Binds PDZ-domain containing proteins. The C-terminal motif is found primarily in avian isolates (common variants include av=ESEV/EPEV/KSEV, hu=RSKV/RSEV). Note: RSKV/RSEV is not	PubMed: 16439620, 20702615, 18334632, 20410267,
NEP	83	V	I		

[a]Sequence functional features in segments were identified using Influenza Research Datebase (IRD, https://www.fludb.org/)

China National Accreditation Service for Conformity Assessment

LABORATORY ACCREDITATION CERTIFICATE

(Registration No. CNAS BL0034)

BSL-3 Laboratory, Institute of Microbiology, Chinese Academy of Sciences

Block 3, No.1, Beichen West Road, Chaoyang District, Beijing, China

is accredited to CNAS-CL05:2009 Accreditation Criteria for Laboratory Bio-safety (Including GB19489:2008 Laboratories —General Requirements for Bio-safety and the relevant requirements of Regulations on Administration of Bio-safety in Pathogenic Microorganism Laboratories) as ABSL-3 laboratory.

It is a laboratory working on conventional amount of pathogenic bio-agents which transmit through aerosols under the condition of availably using primary bio-safety isolation equipments in all procedures.

Date of Issue：2017-08-04

Date of Expiry：2022-08-03

Date of Initial Accreditation：2012-04-20

Signed on behalf of China National Accreditation Service for Conformity Assessment

China National Accreditation Service for Conformity Assessment (CNAS) is authorized by Certification and Accreditation Administration of the People' s Republic of China (CNCA) to operate the national accreditation schemes for conformity assessment. CNAS is a member of the International Laboratory Accreditation Cooperation (ILAC) and the Asia Pacific Laboratory Accreditation Cooperation (APLAC).
